# Phylogenetic Analyses and GAGA-Motif Binding Studies of BBR/BPC Proteins Lend to Clues in GAGA-Motif Recognition and a Regulatory Role in Brassinosteroid Signaling

**DOI:** 10.3389/fpls.2019.00466

**Published:** 2019-04-16

**Authors:** Marius L. Theune, Ulrich Bloss, Luise H. Brand, Friederike Ladwig, Dierk Wanke

**Affiliations:** ^1^Molecular Plant Biology, Saarland University, Saarbrücken, Germany; ^2^ZMBP-Plant Physiology, Tübingen University, Tübingen, Germany

**Keywords:** BBR/BPC proteins, GAGA-factors (GAF), GAGA-binding domain, basic Pentacysteine transcription factors, PRE, Polycomb repressive complexes

## Abstract

Plant GAGA-motif binding factors are encoded by the BARLEY B RECOMBINANT / BASIC PENTACYSTEINE (BBR/BPC) family, which fulfill indispensable functions in growth and development. BBR/BPC proteins control flower development, size of the stem cell niche and seed development through transcriptional regulation of homeotic transcription factor genes. They are responsible for the context dependent recruitment of Polycomb repressive complexes (PRC) or other repressive proteins to GAGA-motifs, which are contained in Polycomb repressive DNA-elements (PREs). Hallmark of the protein family is the highly conserved BPC domain, which is required for DNA binding. Here we study the evolution and diversification of the BBR/BPC family and its DNA-binding domain. Our analyses supports a further division of the family into four main groups (I–IV) and several subgroups, to resolve a strict monophyletic descent of the BPC domain. We prove a polyphyletic origin for group III proteins, which evolved from group I and II members through extensive loss of domains in the *N*-terminus. Conserved motif searches lend to the identification of a WAR/KHGTN consensus and a TIR/K motif at the very *C*-terminus of the BPC-domain. We could show by DPI-ELISA that this signature is required for DNA-binding in AtBPC1. Additional binding studies with AtBPC1, AtBPC6 and mutated oligonucleotides consolidated the binding to GAGA tetramers. To validate these findings, we used previously published ChIP-seq data from GFP-BPC6. We uncovered that many genes of the brassinosteroid signaling pathway are targeted by AtBPC6. Consistently, *bpc6, bpc4 bpc6*, and *lhp1 bpc4 bpc4* mutants display brassinosteroid-dependent root growth phenotypes. Both, a function in brassinosteroid signaling and our phylogenetic data supports a link between BBR/BPC diversification in the land plant lineage and the complexity of flower and seed plant evolution.

## Introduction

GAGA-motif binding factors (GAFs) have an indispensable function for proper growth and development in many multicellular organisms. In animals, Trithorax-like (Trl) and Pipsqueak (Psq) protein families are GAFs that affect gene expression by DNA-looping or by physical interaction with histone-modifying complexes at Polycomb repressive DNA-elements (PREs) (Mishra et al., 2003; Mulholland et al., 2003; Salvaing et al., 2003; Lehmann, 2004; Adkins et al., 2006; Ogiyama et al., 2018).

The plant specific BARLEY B-RECOMBINANT/BASIC PENTACYSTEINE (BBR/BPC) protein family shows also binding preference to GAGA-DNA motifs (Sangwan and O’Brian, 2002; Santi et al., 2003; Meister et al., 2004). Although animal and plant proteins constitute unrelated protein families (Lang et al., 2010; Wanke et al., 2011), it was proposed early on that they might share the same molecular function in PREs (Santi et al., 2003). It is intriguing, though, that animal and plant GAGA-motif binding factors exhibit the same mechanistic function by direct or indirect recruitment of Polycomb Repressive Complex (PRC) members to PREs. These PRCs are well conserved in all eukaryotes and regulate chromatin compaction and gene expression via Histone 3 Lys27 trimethylation (H3K27me3) (Schatlowski et al., 2008; Adrian et al., 2009).

Three groups of BBR/BPC protein are discriminated on the basis of their divergent N-terminus (Meister et al., 2004). In yeast two-hybrid experiments, Arabidopsis BBR/BPC proteins from different groups interacted directly with histone methyltransferases, which are hallmark proteins of the PRC2 (Mu et al., 2017). These data, however, could not be consolidated *in planta* so far, where distinct group wise interactions were observed.

Arabidopsis group II member BPC6 interacts with PRC1 component LIKE-HETEROCHROMATIN PROTEIN 1 (LHP1) *in vivo* to synergistically repress the expression of transcription factor genes *via* H3K27me3 (Hecker et al., 2015). It was shown that BPC6 is required and sufficient to recruit LHP1 to GAGA-motif containing DNA *in vitro*. It was proposed that this ternary DNA-protein-protein complex associates with PRC2 member VERNALIZATION 2 (VRN2) *in vivo*, which subsequently leads to the trimethylation of Histone 3 (Hecker et al., 2015).

Likewise, Arabidopsis group I member BPC1 directly recruits PRC2 components to developmental genes and is thereby directly involved in gene silencing *via* H3K27me3 (Xiao et al., 2017). It was shown that the GAGA motifs contained in PREs are required and sufficient for the repressive function (Xiao et al., 2017).

At least one additional mechanism of repression exists for BPC1 through direct physical interaction with SEUSS (SEU) – LEUNIG (LUG) transcriptional cosuppressors to repress the homeotic *SEEDSTICK* (*STK*) locus (Simonini et al., 2012).

Other mechanism for gene regulation, for example DNA-bending, were also observed for both animal and plant GAFs (Kooiker et al., 2005; Ogiyama et al., 2018). It was proposed that GAFs form higher order complexes to induce these conformational chromatin changes (Lehmann, 2004; Adkins et al., 2006; Ogiyama et al., 2018). In animals, however, GAF-dependent Chromatin looping at PREs is sufficient to induce gene silencing also in the absence of PRC function (Ogiyama et al., 2018). Group I BBR/BPC proteins from Arabidopsis also possess such DNA-bending properties *in vitro* (Kooiker et al., 2005), but it remains elusive whether higher order BBR/BPC complexes form inside the nucleus under native conditions.

Group II BBR/BPC proteins harbor an Alanine-zipper like coiled-coild domain at their N-terminus, which facilitates homotypic dimerization (Wanke et al., 2011). The dimerization forms through electrostatic interaction between salt bridges in parallel oriented coiled-coils. It was proposed that structure and conformation of this domain theoretically allows for possible higher order complex formation (Wanke et al., 2011), but experimental evidence is lacking so far.

Functional analyses revealed an indispensable role for BBR/BPC proteins in gene expression control of transcription factor genes, especially of those with homeotic function (Santi et al., 2003; Kooiker et al., 2005; Berger et al., 2011; Monfared et al., 2011; Winter et al., 2011; Simonini et al., 2012; Simonini and Kater, 2014; Hecker et al., 2015). While loss of function mutants of individual BBR/BPC genes did not lead to significant phenotypic alterations, the analysis of Arabidopsis *bpc1,2,3,4,6* quintuple mutant lines disclosed a function for BBR/BPC proteins in Ethylene and cytokinin phytohormone signaling (Monfared et al., 2011; Shanks et al., 2018).

The hallmark of the entire BBR/BPC family is the evolutionary conserved Basic PentaCysteine (BPC) DNA-binding domain at the very C-terminus of the proteins (Sangwan and O’Brian, 2002; Santi et al., 2003; Meister et al., 2004; Kooiker et al., 2005; Wanke et al., 2011). This domain contains five highly conserved Cysteine residues that were proposed to form a novel type of zinc-finger structure, which is directly involved in the DNA-binding process (Santi et al., 2003; Meister et al., 2004; Kooiker et al., 2005). A recent study revised this idea and proposed an indispensable function for the five Cysteines in dimerization and stabilization of the protein structure by disulfide bonds (Theune et al., 2017). While oxidizing conditions abolished DNA-binding, excess or depletion of zinc ions did not affect binding of Arabidopsis BPC1 to GAGA-motif containing DNA probes *in vitro* (Theune et al., 2017), which confirms an indirect and rather dispensable function for the five Cysteines in GAGA-recognition. Consistently, covalent inter- and intramolecular S-S bonds readily explain the strong interactions between BBR/BPC members, which were observed in previous experiments, for example in gel shift assays (Meister et al., 2004; Kooiker et al., 2005; Theune et al., 2017).

Although BBR/BPC proteins bind to extended GA/TC dinucleotide repeats *in vivo* (Xiao et al., 2017; Shanks et al., 2018), it was proposed that the minimal requirement for proper DNA-binding is a GAGA/TCTC tetranucleotide consensus (Brand et al., 2010; Fischer et al., 2016). Hence, more BBR/BPC proteins bound to neighboring or partially overlapping GAGA tetranucleotides might explain the observed preference for longer motifs *in vivo*. Genome wide motif distribution analyses in Arabidopsis and rice uncovered an orientation dependent enrichment of extended GAGA-motifs close to the transcription start site and in introns (Santi et al., 2003; Berendzen et al., 2006; Hecker et al., 2015).

To gain an insight into the evolutionary history of BBR/BPC proteins, we compiled phylogenetic trees based on the conserved BPC-domain and the full-length protein sequences. Our analyses not only uncovered additional groups and subgroups of BBR/BPC proteins, but also lend to structural clues on DNA-binding. We could show that conserved amino acid motifs at the very C-terminus of Arabidopsis BPC1 are important for successful GAGA-motif recognition. DNA-binding studies with Arabidopsis BPC1 and BPC6 confirmed the binding to GAGA tetranucleotide motifs. Analysis of previously published ChIP-seq data (Shanks et al., 2018) showed that Arabidopsis BPC6 targets the promoters of all major brassinosteroid-signaling components. A root-growth assay with different concentrations of the phytohormone Brassinolide consolidated a role for BPC6 in brassinosteroid signaling.

## Results

### Diversification of the BPC Domain

**FIGURE 1:**
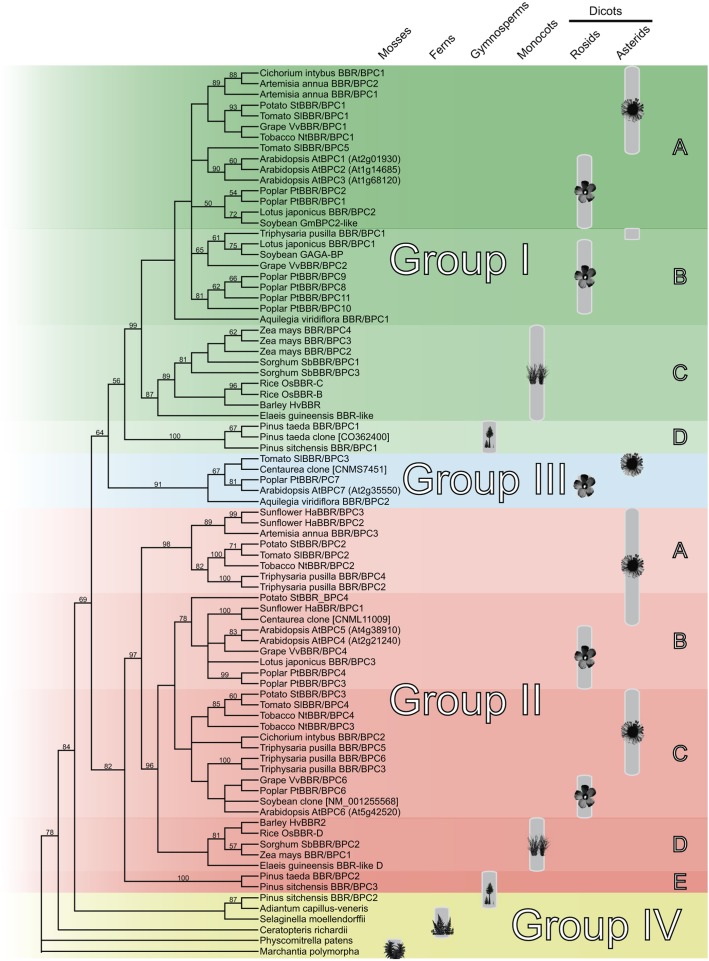
Cladogram of the monophyletic Basic PentaCysteine (BPC) DNA-binding domain. Neighbor-Joining tree based on an alignment of 83 BPC domain protein sequences. The plant symbols indicate the distribution of the BPC proteins all over the vascular plants with maturing diversification in higher dicots. The tree topology was computed using phylogeny.fr with 1000 bootstrap replications and Jones-Taylor-Thornton matrix distances. Only bootstrap values above 50% are shown. Nodes with bootstrap values below 20% are collapsed.

The Basic PentaCysteine DNA-binding domain represents the sole characteristic feature shared by all BBR/BPC family members. To gain an insight into its diversification in plant evolution, we first computed a cladogram on the basis of the BPC domain. The high degree of conservation in sequence and position at the very *C*-terminus of the proteins allowed us to identify 83 high confidence BPC domains for our analyses, which are 94 – 104 amino acids in size Supplementary Table S1. These sequences cover representative species of all major land plant phyla, such as mosses, ferns, gymnosperms and angiosperms. The cladogram nicely resolves the monophyletic origin of the BPC domain and the already known major clades of groups I and II (Figure 1). The divergence of group I and II BPC domains occurred prior to gymnosperms, because both clades are restricted to seed plants. Closer inspection of group III does not disclose a monophylum, but proposes a polyphyletic origin of the representative domains. Besides the three groups of known BBR/BPC proteins, we discovered a fourth group with only basal plant species and in which angiosperm representatives are lacking. This discrimination of group IV is justified not only by sequence, but also by the presence of three clades of gymnosperm domains that now reside in group I, II and novel group IV. In addition, the cladogram gives support to further refinement of group I and II domains into subgroups, because several independent clades with sister groups resolved within the seed plants (Figure 1). Differentiation of subgroups results in gymnosperm (ID and IIE) or monocot (IC and IID) specific clades. Groups IA and IB as well as groups IIB and IIC contain both rosids and asterids representatives, which otherwise would reside in large paraphyletic groups I and II. Consequently, an asterids specific subgroup IIA is clearly separated from the other asterids containing clades within group II.

Interestingly, no BPC domains were discovered outside the land plant lineage so far, for example in *Ostreococcus, Chara* or any other green algae, which supports the idea of an important function for BBR/BPC proteins that is restricted to land plants (Lang et al., 2010).

### Diversification and Evolution of the BBR/BPC Family

**FIGURE 2:**
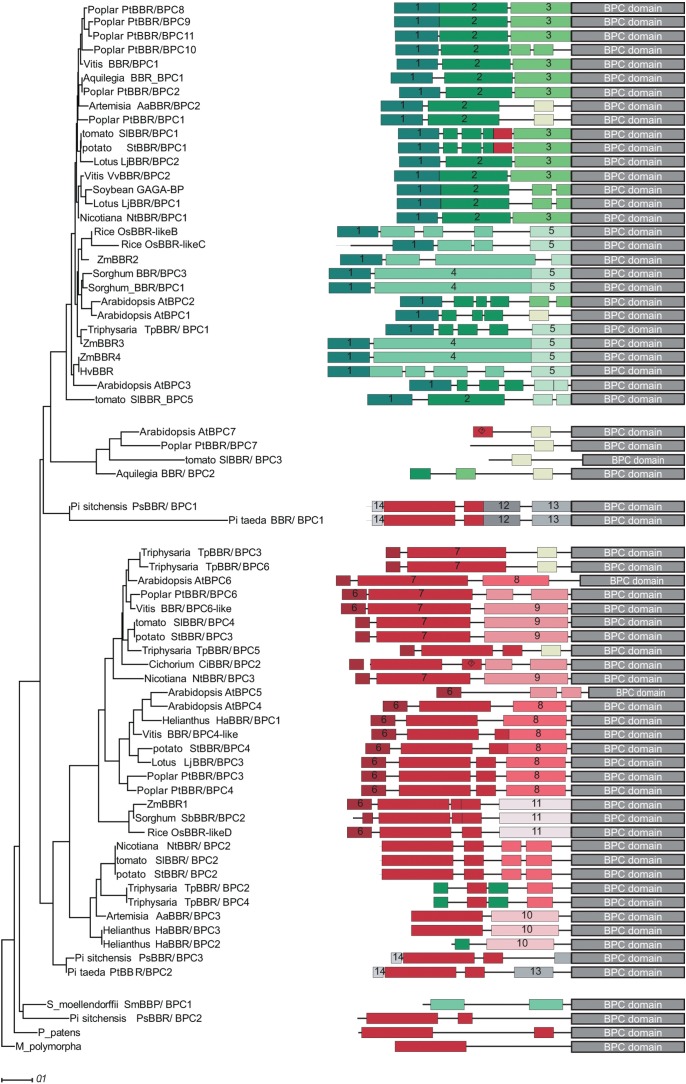
Phylogram and conserved domain structures of full-length BBR/BPC proteins. The phylogram (left) and the corresponding protein domain structures (right) are based of 68 full-length BBR/BPC protein sequences. The phylogenetic tree was computed using phylogeny.fr (Dereeper et al., 2008) with 1000 bootstrap replications and Jones-Taylor-Thornton matrix distances. Only bootstrap values above 50% are shown. Nodes with bootstrap values below 20% are collapsed. Conserved domains were identified using MEME software suit^2^ tool collection. A motif overview is provided as Supplementary Data Sheet S1.

We next computed a phylogenetic tree with 68 full-length protein sequences Supplementary Table S2, which supports the groups and subgroups that we identified solely on the basis of the BPC domain (Figure 2). Gymnosperm BBR/BPC sequences nicely delimitate the groups and are located at the base of group I and group II branches, and at the top of group IV. Group III proteins are embraced by group I members, which contradicts their status as an independent group and validates their polyphyletic origin.

To investigate a possible gain or loss of domains and conserved motifs outside the conserved BPC domain, which are characteristic for groups or subgroups, a motif discovery analysis was performed (Figure 2). A total of 14 protein motifs were identified that met our requirements (Supplementary Data Sheet S2). BBR/BPC proteins in dicot species exhibit a rather conservative domain arrangement, irrespective of the groups: The *N*-terminus of group I proteins is composed of consecutive motifs 1, 2, and 3. Likewise, a typical group II protein is characterized by the highly conserved peptide motif 7. The alanine zipper like coiled-coil dimerization domain, which is also required for interaction with LHP1 (Wanke et al., 2011; Hecker et al., 2015), is contained in motif 6. Interestingly, motif 4 is characteristic for monocot specific subgroup IC and as ancestral partial signature in individual group II and IV members. Similarly, a partial signature of group II hallmark motif 7 is found in group IA. The presence of these two partial signatures in converse groups might be indicative of a certain domain rearrangement. The only motif shared between group IV members is an ancestral form of motif 7, which is truncated at its N-terminus. It is most noteworthy that group III members contain truncated motifs of different groups, which can be explained by extensive loss of domains throughout evolutionary time and, again, supports its polyphyletic origin. Another important observation is that Arabidopsis BBR/BPC proteins have a tendency to be shorter than their nearest orthologs, due to missing or fragmented motif composition. Especially, group I proteins from Arabidopsis do not contain the archetype motif composition of other group I BBR/BPCs, but lack motifs 2 and 3 of dicot or motifs 4 and 5 of monocot proteins.

A closer inspection of the sequences revealed short arginine and lysine rich peptides in motifs 4, 7, 9, 10, 11, 12, 13, and 14, which might serve as nuclear localization signals (NLS). Motif 4 is specific to the monocot species in group IC and rich in histidine residues of unknown function, a feature that was already noted before (Santi et al., 2003).

None of the conserved peptide motifs displayed a significant similarity to proteins outside the land plant lineage, besides the Alanine-zipper signature that is localized at the very *N*-terminus of group II and some group IV BBR/BPC members. It was reported before that these Alanine-zipper like motifs are common in different protein families, especially from the eukaryote phylum (Wanke et al., 2011).

### Intron- and Exon Structure of Selected *BBR*/*BPC* Genes

**FIGURE 3:**
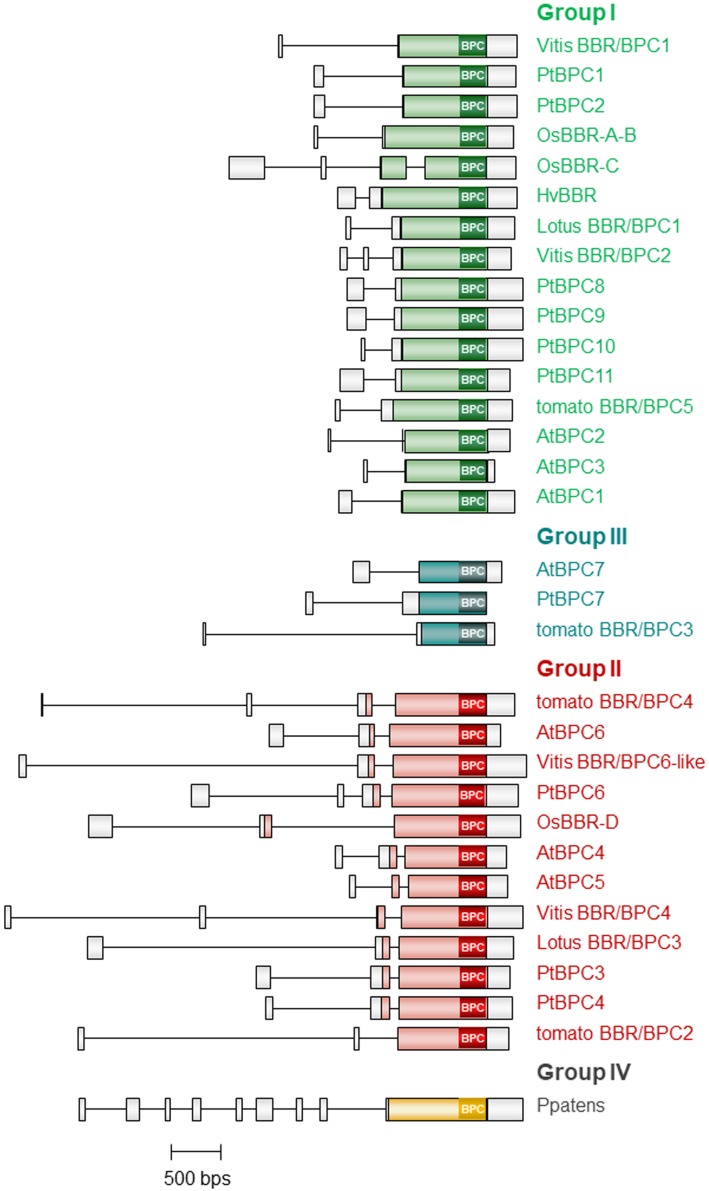
Intron- and Exon Structure of selected BBR/BPC genes. Schematic representation of exons (boxes) and introns (lines) in selected members of the BBR/BPC proteins. Coding regions of the different groups are color-coded.

With full-length sequences at hand, we were able to display the intron-exon structure for selected *BBR*/*BPC* genes, where genomic sequences were available (Figure 3). All *BBR*/*BPC* genes possess 5′UTR introns, which are shortest in group I and largest in basal group IV genes. The 5′UTR of the basal group IV gene from the moss *Physcomitrella patens* is characterized by multiple introns and exons. The coding sequences of group II genes contain a short intron in the 5′ part, which is missing in all other groups, where the coding sequence is not interrupted by introns.

### High Degree of Conservation in the BPC Domain

**FIGURE 4:**
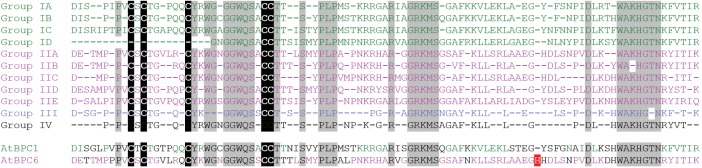
High degree of conservation in the BPC domain. Protein sequence alignment of the Basic PentaCysteine (BPC) DNA-binding domain consensi, that were derived from protein sequences contained in Supplementary Data Sheet S1. Positions that are evolutionary retained in all BBR/BPC group members are highlighted by gray background The highly conserved Cysteines are emphasized by black background. The unique histidine residue that occurs only in Arabidopsis BPC6 is indicated in red.

Although many researchers analyzed the DNA-binding capacity of the BPC domain, the region responsible for GAGA-motif recognition was not discovered so far. To identify conserved residues that might be involved in direct DNA contact (Supplementary Data Sheet S1), we derived consensus sequences with invariant amino acids for each of the subgroups (Figure 4). It became evident that three regions with obviously higher degree in conservation exist: One, the section surrounding the five conserved cysteines. Two, a central piece with an invariant RxxGRKMS motif. Three, a C-proximal WAR/KHGTN consensus.

In addition, we observed that Arabidopsis BPC6 was the only BBR/BPC protein with an aberrant spacing between the conserved consensus sequences: An extra histidine residue is inserted between the second and third conserved motif, which might possibly affect the domain functions (Figure 4). However, GAGA-motif recognition between BPC1 or BPC6 was not significantly different (Meister et al., 2004; Brand et al., 2010; Fischer et al., 2016; Theune et al., 2017). Hence, the extra histidine residue must be located at a position where it is not in conflict with DNA-binding, dimerization of the domain or any other function. We next used the atomic coordinates of an *ab initio* computer model structure of the Arabidopsis BPC1 DNA-binding domain (Theune et al., 2017), to derive a model structure for BPC6. A comparison of the two BPC-domain models suggest that all conserved positions are freely accessible and located at the domain’s surface (Supplementary Image S1). Also the extra histidine residue within the BPC6 domain is predicted at the surface of the domain, but not close to any of the conserved regions (Supplementary Image S1).

### The Conserved WAR/KHGTN Motif Is Required for DNA-Binding

The predicted model structures of the BPC1 DNA-binding domain suggested that the *C*-proximal WARHGTN motif possibly protrudes from a globular domain and, hence, constitutes a better candidate for DNA-binding than the central GRKMS motif. In addition, we decided to focus on the WAR/KHGTN consensus first, simply because of the ease to truncate the *C*-terminus.

**FIGURE 5:**
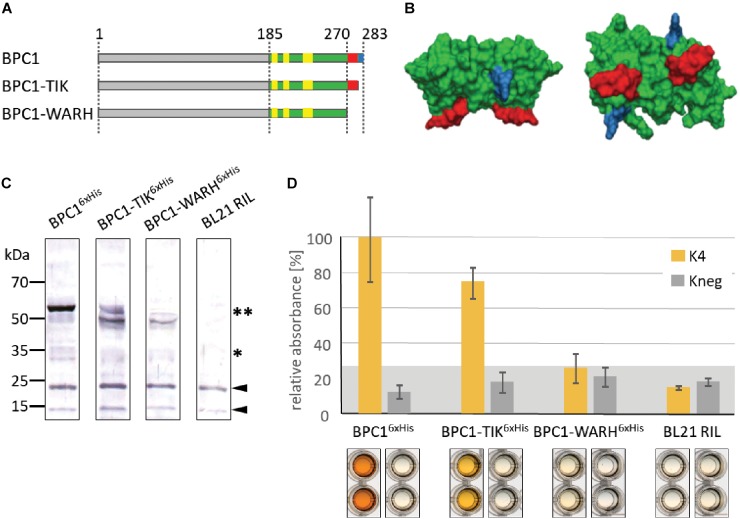
Binding capacity of BPC1 mutants. **(A)** Schematic overview of all 6×His-epitope tagged BPC1 mutants and truncations. The highly conserved Cysteines are highlighted by yellow boxes. The position of the conserved WARHGTN signature is indicated (red); the final three amino acids consensus TIR is indicated in blue color. **(B)** Structural model of a putative BPC1 domain dimer to illustrate predicted positions for conserved amino acids. The conserved WARHGTN motif is highlighted in red color. The conserved TIR motif at the *C*-terminus is indicated in blue color. **(C)** Gel-blot experiments with immunological detection of all recombinant proteins. The expected molecular weights for monomer (^∗^) and dimer (^∗∗^) proteins are indicated. **(D)** Specific binding of 6×His-epitope tagged BPC1 versions to positive (K4) and negative (Kneg) dsDNA-probes in DPI-ELISA experiments. The histogram bars show normalized signal intensities and error bars represent one standard deviation. Gray background shading indicates level of confidence for significant binding (*t*-test *p* < 0.05).

We constructed two truncated versions of BPC1 and tested them by DNA-protein interaction (DPI)-ELISA for binding to GAGA-containing dsDNA-probes (Figure 5A). A dimer model structure of the DNA-binding domain for BPC1 illustrates where the conserved TIR and WARHGTN motifs are probably positioned (Figure 5B). Consequently, only the final three amino acids were removed in BPC1-TIR proteins, while the last 13 amino acids were deleted in BPC1-WARH. All three BPC1 versions were expressed as recombinant 6× His-tagged proteins (Figure 5C) and tested for their binding capacities in DPI-ELISA experiments (Figure 5D). About 30% less BPC1-TIR did bind to the positive GAGA-containing DNA probe (K4), compared with the wildtype extract. Moreover, the BPC1-WARH protein lost its capabilities to bind to GAGA-motifs, which is consistent with the model structure and underlines the importance of the conserved WAR/KHGTN consensus in DNA-recognition. Given its high degree in conservation, one can propose that all BBR/BPC protein make a possible direct physical contact to DNA through this conserved motif.

### Arabidopsis BPC1 and BPC6 Bind to GAGA/TCTC Tetranucleotides

Even though the binding of BPC1 and BPC6 to genomic target sites validated their affinity to extended GA/TC-repeats *in vivo* (Xiao et al., 2017; Shanks et al., 2018), the minimal motif that is required for BBR/BPC-binding is still under discussion. Initial studies suggested that BBR/BPC proteins require at least dodecamers (GA/TC)_6_ for proper DNA binding (Sangwan and O’Brian, 2002; Santi et al., 2003; Meister et al., 2004). The groups of Colombo, Gasser, and Kater published yeast one-hybrid data and extensive EMSA competition assays that lend to the identification of at least a nonanucleotide RGARAGRRA binding consensus as minimal requirement for BBR/BPC binding (Kooiker et al., 2005; Monfared et al., 2011; Simonini et al., 2012; Simonini and Kater, 2014). Moreover, the shift assays of these groups uncovered multiple bands that strongly imply the binding of BBR/BPC-dimers/multimers to a singly oligonucleotide probe (Meister et al., 2004; Kooiker et al., 2005). Data from our group suggest a binding of BBR/BPC proteins to short GAGA/TCTC tetranucleotide motifs, which was analyzed predominantly by classical DPI-ELISA and quantitative qDPI-ELISA experiments (Brand et al., 2010; Hecker et al., 2015; Fischer et al., 2016).

**FIGURE 6:**
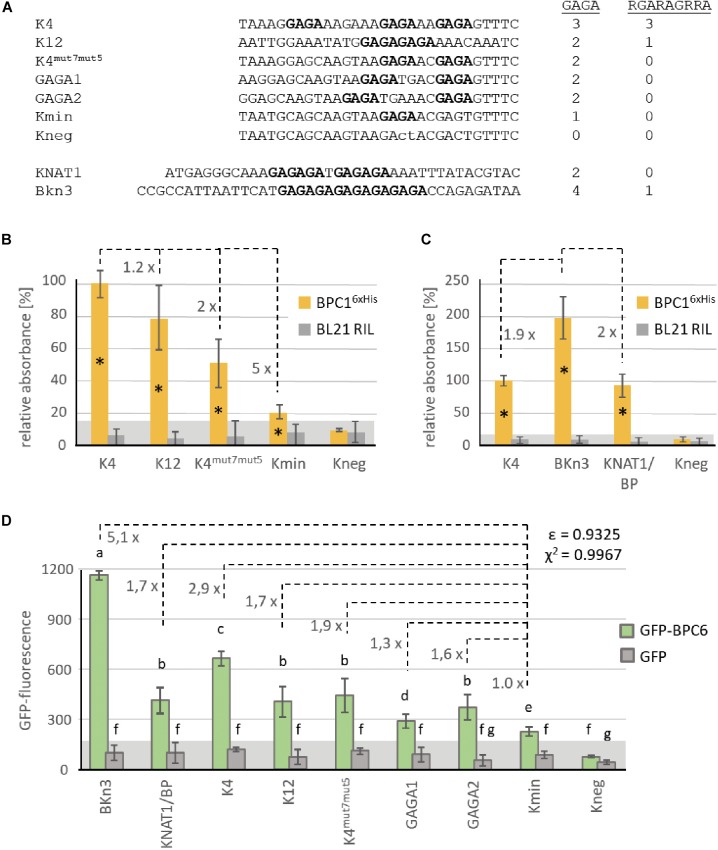
Comparative binding studies with BPC1 and BPC6. **(A)** DNA-sequences of the double stranded oligonucleotide probes. Only the biotinylated sense strand is given in 5′ to 3′-orientation. GAGA/TCTC tetranucleotides are highlighted in bold. The number of GAGA- or RGARAGRRA-motifs contained in each DNA-probe is indicated. **(B,C)** Specific binding of 6×His-epitope tagged BPC1 to the indicated double stranded oligonucleotide probes. Data of both histograms were derived from different experiments with different protein extracts. The histogram bars show normalized signal intensities that relate to the binding intensity with the K4 probe. The fold difference between the signal intensities of different bars are indicated at a dashed line. Error bars represent one standard deviation. Gray background shading indicates level of confidence for significant binding (*t*-test *p* < 0.05). **(D)** Specific binding of recombinant GFP-BPC6 to indicated DNA-probes in qDPI-ELISA experiments. Histogram bars are derived from data from different experiments with three different extracts and show raw GFP-fluorescence. Numbers at dashed lines indicate the fold difference between the fluorescence signals of different probes related to the Kmin probe, with only s single GAGA-motif. Error bars represent one standard deviation. Gray background shading indicates level of confidence for significant binding (*t*-test *p* < 0.05). The correlation between the number of GAGA-tetranucleotide motifs and the fold difference to Kmin is indicated as Pearson ε or as χ^2^.

To tackle the issue of minimal requirement for binding, we derived several double-stranded oligonucleotide probes from previously published articles for testing in DPI-ELISA (Figure 6A): Kooiker and colleagues demonstrated *in vitro* binding of BPC1 to elements 4 and 12 of the *SEEDSTICK* (*STK*) promotor region (Kooiker et al., 2005), here termed K4 and K12, respectively. The K4 oligonucleotide was chosen for further mutations that consecutively reduced the number of possible non-overlapping GAGA/TCTC motifs from 3× GAGA (K4), 2× GAGA (K4^mut7mut5^), 1× GAGA (Kmin) to 0× GAGA (Kneg). To generate K4^mut7mut5^, we replaced single nucleotides in K4, which were already known to be non-binding mutations (Kooiker et al., 2005). We also generated two oligonucleotide probes, GAGA1 and GAGA2, based on the K4^mut7mut5^ sequence that contain the two GAGA motifs with different spacing. In addition, BPC1 was shown to target a promotor region of the *KNAT1*/*BREVIPEDICELLUS* (*BP*) gene (Simonini and Kater, 2014), which was termed KNAT1/BP in our study. We also included the (GA/TC)_8_ element of the barley *Knotted* 3 (*BKn3*) gene, which is bound by the group I BBR protein (Santi et al., 2003). As these oligonucleotide probes differ in number and spacing of non-overlapping GAGA or RGARAGRRA motifs (Figure 6A), we assumed that quantitative differences in binding capacities will be uncovered, which might correlate with either the degenerate longer or the shorter DNA-binding motif.

Indeed, the signal intensities with equal amount of recombinant BPC1^6×His^ decreased consecutively with the decrease in available binding sites (Figure 6B). It is noteworthy, that the oligonucleotides K4^mut7mut5^ and Kmin displayed a significant signal over the background, which is indicative for BPC1^6×His^ binding, although these probes do not contain a RGARAGRRA consensus (Figure 6A). While K4 and KNAT1/BP are bound with a comparable amount of BPC1^6×His^, signal intensities for the BKn3 oligonucleotide were twice as high (Figure 6C). These data imply that twice as many BPC1^6×His^ can bind to a (GA/TC)_8_ repeat compared with K4 or KNAT1/BP. The DPI-ELISA provides only a semi-quantitative measure, because signal intensities depend on the enzymatic antibody detection and increase over time until saturated (Brand et al., 2010, 2013b; Fischer et al., 2016). Hence, it is not surprising that the ratio between the signal intensities does not exactly reflect the motif abundance.

We, therefore, decided to repeat the binding studies with selected oligonucleotides in a qDPI-ELISA with recombinant GFP-BPC6 and GFP, as a control. Previous experiments with GFP-BPC6 disclosed that the qDPI-ELISA allows a near quantitative analysis of the binding capacities of a protein, which is mainly due to the fluorometric detection (Fischer et al., 2016). In general, the GFP-BPC6 fluorescence for the different oligonucleotides was comparable to the signal intensities with BPC1^6×His^ (Figure 6D). The DNA-probes KNAT1/BP, K12, K4^mut7mut5^ and GAGA2 with two non-overlapping GAGA/TCTC motifs display fluorescence signals with similar intensities (Figure 6D). The result for GAGA1, which also contains 2x GAGA/TCTC, is less than expected and intermediate between its related oligonucleotides GAGA2 and Kmin (Figure 6D).

Next, we wanted to compare the signal intensities of GFP-BPC6 with the known availability of non-overlapping binding motifs. Therefore, the background fluorescence of the negative binding probe Kneg was subtracted from the GFP-fluorescence value of all other oligonucleotide probes (Supplementary Table S3). The fold difference was calculated to the base of Kmin, which contains only a single GAGA/TCTC motif (Figure 6A). We now computed the Pearson correlation coefficient ε and the χ^2^ based on the fold differences and the presence of the different motifs. Although there was a positive Pearson correlation between the number of motifs and fluorescence intensity for both motifs, the coefficient was much higher for the GAGA-motif (ε = 0.9325) than the RGARAGRRA (ε = 0.52167) (Figure 6D and Supplementary Table S3). Even clearer was the result for the χ^2^ based analysis, where the signal intensities cannot be explained by the presence of the RGARAGRRA consensus (χ^2^ = 0.1785) (Supplementary Table S3). In contrast, there is a high interdependency between the fluorescence and the number GAGA/TCTC motifs (χ^2^ = 0.9967). Hence, the presence of a tetranucleotide GAGA-site is sufficient and can almost fully explain the binding preference in our test series.

Interestingly, signal intensities were higher than expected for the BKn3 oligonucleotide probe that contains a (GA/TC)_8_ repeat of four non-overlapping GAGA/TCTC motifs (Figure 6C, D). For example, fluorescence signals of the KNAT1/BP probe, where a longer GA/TC-repeat is split into two GAGAGA hexanucleotides by a single base-pair insertion, were 2.5-times lower than that of BKn3. These findings imply, that longer consecutive GA/TC-repeats might allow for partial overlapping binding of BBR/BPC proteins or that the minimal binding site might even be shorter than a tetramer.

### Arabidopsis BPC6 Targets the Brassinosteroid Hormone Pathway

**FIGURE 7:**
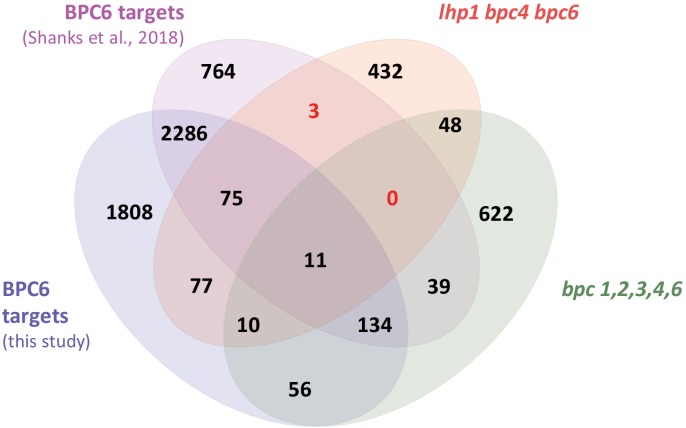
Overlap between the two different BPC6 target gene lists and the transcriptome in bpc mutants. Venn diagram display of the BPC6 target genes from our analysis, the BPC6 target genes from a previous publication (Shanks et al., 2018) and the transcriptome of mutants that are impaired in *BPC6* function. Significantly underrepresented overlap is indicated in red.

With the quantitative binding information for GFP-BPC6 at hand, we intended to link these findings with *in vivo* data. We therefore mined the publically available ChIP-sequencing dataset for GFP-BPC6 and compared it with the transcriptome of *bpc1,2,3,4,6* roots (Shanks et al., 2018). To our surprise, there was only little overlap between the proposed BPC6 *in vivo* targets and the genes deregulated in *bpc1,2,3,4,6*-mutant plants (Figure 7). We therefore reanalyzed the dataset and now consider 5873 BPC6-target regions close to an annotated gene for further analyses (Supplementary Table S4). For reasons of intercomparability with previously published transcriptome data, we restrict our analyses to those 4457 target genes present on the ATH1 gene chip. Next, we compared our set of BPC6 target genes with the transcriptome data of *lhp1 bpc4 bpc6* (Hecker et al., 2015) and *bpc1,2,3,4,6* (Shanks et al., 2018), as well as the previously analyzed BPC6 targets (Shanks et al., 2018). As expected, a considerable amount of genes was shared between both analyses of the BPC6-targets (Figure 7). Nevertheless, 40-50% of the *in vivo* targets were specific for only one of the analyses. It is noteworthy that 363 genes from our reanalyzed BPC6-target gene set were also differentially expressed in the *lhp1 bpc4 bpc6* seedlings or *bpc1,2,3,4,6* mutant roots (Figure 7). Of those BPC6 targets from the previous publication (Shanks et al., 2018), which did not overlap with our reanalyzed target gene list, only a significantly little portion of 42 genes overlapped with the differentially expressed genes in both mutants. Mainly because of this disproportional overlap of the previous target gene list with the transcriptome data, we decided to focus on our reanalyzed BPC6 target gene dataset for further analyses.

We next wanted to identify transcriptional responses, which depend on BPC6 *in vivo* binding. As a previous contributor to the multinational AtGenExpress collaborative (Kilian et al., 2007), we decided to mine this data repository for considerable overlap between our BPC6 target gene list and differentially expressed genes from other experiments. As all experiments of the AtGenExpress project make use of same ATH1 gene chip platform, this transcriptome data provides a very high degree of robustness and intercomparability (Kilian et al., 2012). Moreover, this unique repository allows us to compare our BPC6 target genes as an input with thousands of gene lists that were derived from more than 1000 experiments and, hence, provides an invaluable resource for information.

**FIGURE 8:**
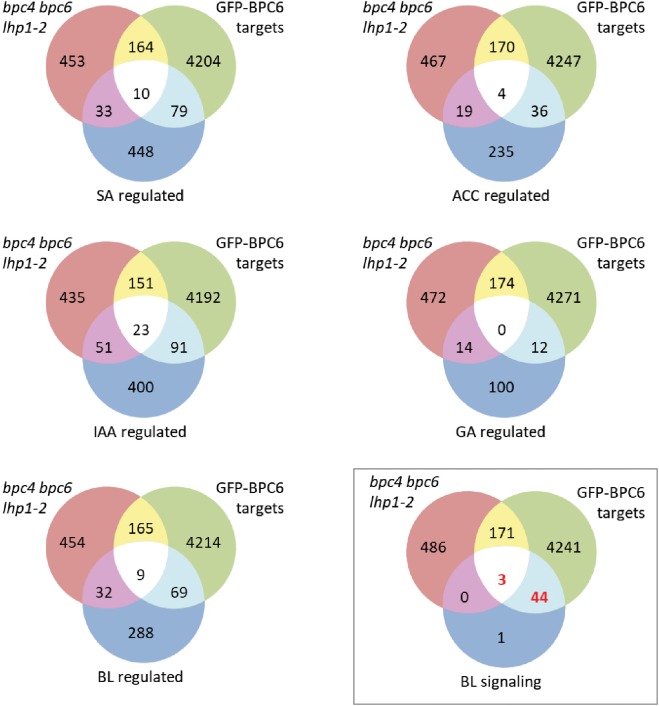
Comparison of our BPC6 target genes with selected phytohormone gene lists. Venn diagram comparison of the indicated gene lists with our BPC6 target gene list and the differentially expressed genes in the *lhp1 bpc4 bpc6* mutant (Hecker et al., 2015). The gene lists are provided as Supplementary Table S7. The comparison with the 48 brassinosteroid signaling genes is highlighted by a box. Significantly enriched overlap is indicated in red.

The largest and most significant overlap was found between our BPC6 target gene list and a hand edited list of 48 genes involved in brassinosteroid signaling (Figure 8 and Supplementary Table S5). This is an interesting observation, because of the already known linkage of BBR/BPC proteins with ethylene and cytokinin responses (Monfared et al., 2011; Shanks et al., 2018). A comparison with other lists of genes that are responding to Brassinolide or other phytohormone treatments did not uncover a significant overlap (Figure 8).

**FIGURE 9:**
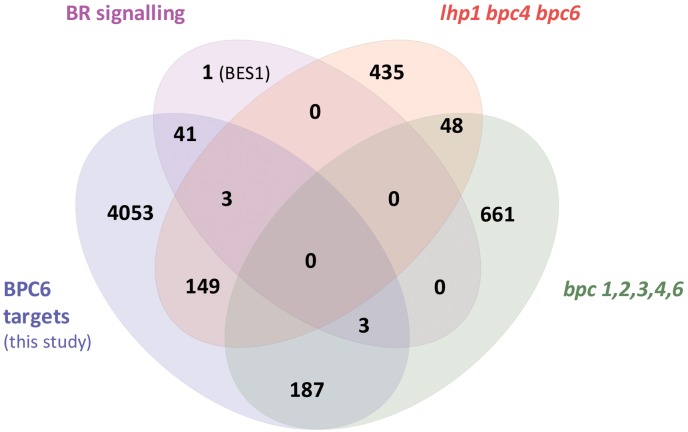
Only six brassinosteroid signaling genes are differentially expressed in mutants plants. Overlap between our BPC6 target gene list, the 48 brassinosteroid signaling genes and the differentially expressed genes in *lhp1 bpc4 bpc6* or in *bpc1,2,3,4,6* mutant roots.

We next compared the expression of the brassinosteroid signaling gene targets with genes deregulated in *lhp1 bpc4 bpc6* seedlings or in the roots of *bpc1,2,3,4,6* (Supplementary Table S6) (Shanks et al., 2018). The majority of 41 genes were not differentially expressed in any of the mutants. Only *WRKY40, HBI1* and *PRE1* were deregulated in *lhp1 bpc4 bpc6*, while *BIL1, SERK2* and *MYBL2* were differentially expressed in the *bpc1,2,3,4,6* mutant (Figure 9).

**FIGURE 10:**
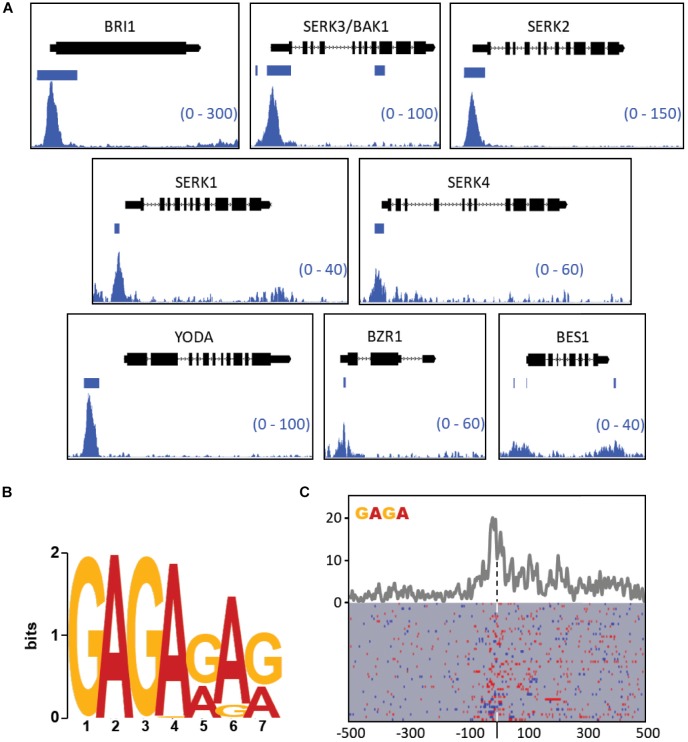
Visualization of GFP-BPC6 binding sites upstream of central genes involved in the brassinosteroid signaling. **(A)** BPC6 binding sites upstream of multiple genes involved in the brassinosteroid response are visualized by using the Integrative Genome Browser. The bedgraphs for GFP-BPC6 ChIP binding sites are shown as two merged biological replicates. Regions of more than 5-fold enrichment over the GFP control is indicated a horizontal bar. The gene models are shown above each panel, with boxes corresponding to exons and lines to introns, with the direction of transcription indicated by small arrows. The shown data range for each investigated gene is shown in the bottom right corner. **(B)** Identification of most highly enriched sequence logo in the promotor of the 48 brassinosteroid signaling genes. **(C)** Motif density map (top) and distribution map (bottom) of the tetranucleotide GAGA (blue) or TCTC (red) in the brassinosteroid signaling genes. The maps are centered (dashed line) at the highest peak in the bedgraph data Supplementary Table S8. A detailed map is provided as Supplementary Data Sheet S4.

BES1, a major integrator of brassinosteroid responses, was the sole signaling component of the list that was not targeted by BPC6 with significance. At closer visual inspection of the data, we found that BPC6 binds to the promoter region of all the 48 brassinosteroid signaling components (Figure 10A and Supplementary Data Sheet S3). Also at the *BES1* locus a more than 5-fold difference from the controls in both replicates can be observed, which is an indication of a weak or transient binding of BPC6 in the promotor and in the 3′UTR.

Most other target genes exhibit strong and defined peaks at neighboring GAGA motifs. For example, the brassinosteroid receptor gene *BRI1* as well as its coreceptors *SERK1, SERK2, BAK1*/*SERK3*, and *SERK4* show pronounced enrichments close to the transcription start. Other genes do not exhibit such distinct enrichment, but are still significantly targeted by BPC6 (Supplementary Data Sheet S3). Interestingly, we observed that the identical GAGAGATGAGAGA motif is contained in the promoters of *BAK1* and *KNAT1/BP*. Consistently, the element in both promoters is an *in vivo* target by BPC6.

To study the DNA-binding properties of BPC6 in this subset of genes, we analyzed the promoters of these 48 brassinosteroid signaling genes for enrichment in cis-elements using the MEME suit (Bailey et al., 2009). Consistent with our expectations, the most prominent motif was a GA/TC-repeat heptanucleotide with an invariant GAGA-tetranucleotide at its beginning (Figure 10B). A density plot of the GAGA/TCTC motifs in the brassinosteroid signaling genes, which is centered to the maximum of the reads in the ChIP-seq data, demonstrates an enrichment of motifs at the center and 3′ toward the gene start (Figure 10C).

**FIGURE 11:**
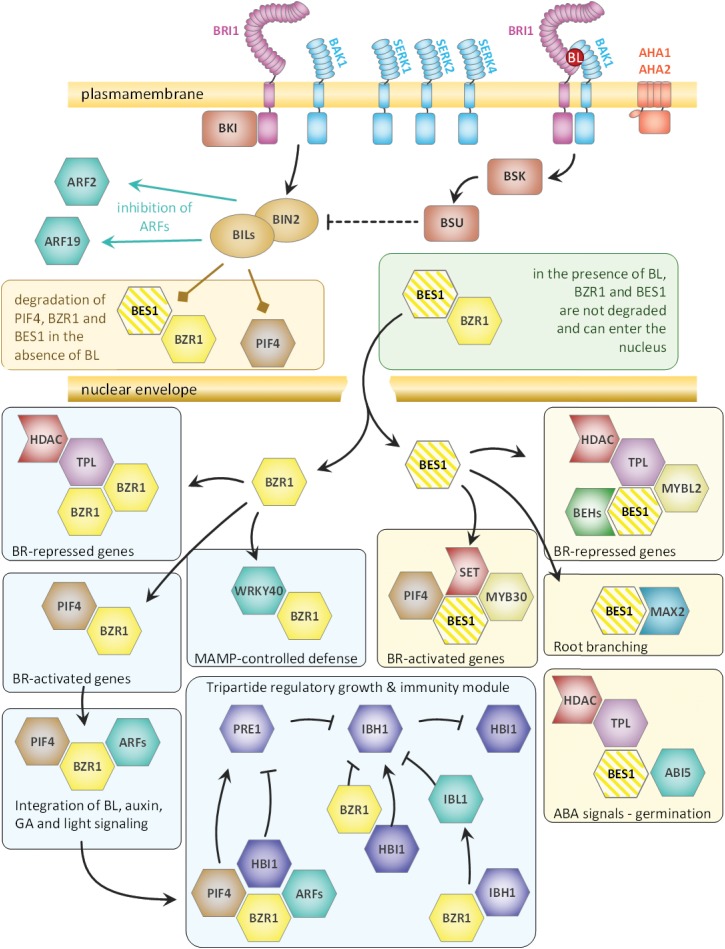
Schematic overview of the brassinosteroid pathway components that are targeted by BPC6 *in vivo*. The composition of the overview is derived from previous publications (Witthoft and Harter, 2011; Belkhadir and Jaillais, 2015; Ladwig et al., 2015). All genes of the brassinosteroid signaling component, except for BES1, are targeted by BPC6 with high confidence. As BPC6 binding to BES1 is only weak, it is colored with stripes.

To give an overview over all the BPC6 target genes that encode brassinosteroid signaling components, we summarized our findings according to previous publications (Witthoft and Harter, 2011; Belkhadir and Jaillais, 2015; Ladwig et al., 2015 and Figure 11). It becomes evident that BPC6 targets the genes of the brassinosteroid signaling pathway at very different levels, which includes all known receptors and co-receptors, all known second messengers as well as the major downstream integrators.

### Mutants Impaired in BPC6 Function Display an Altered Response to Brassinolide

**FIGURE 12:**
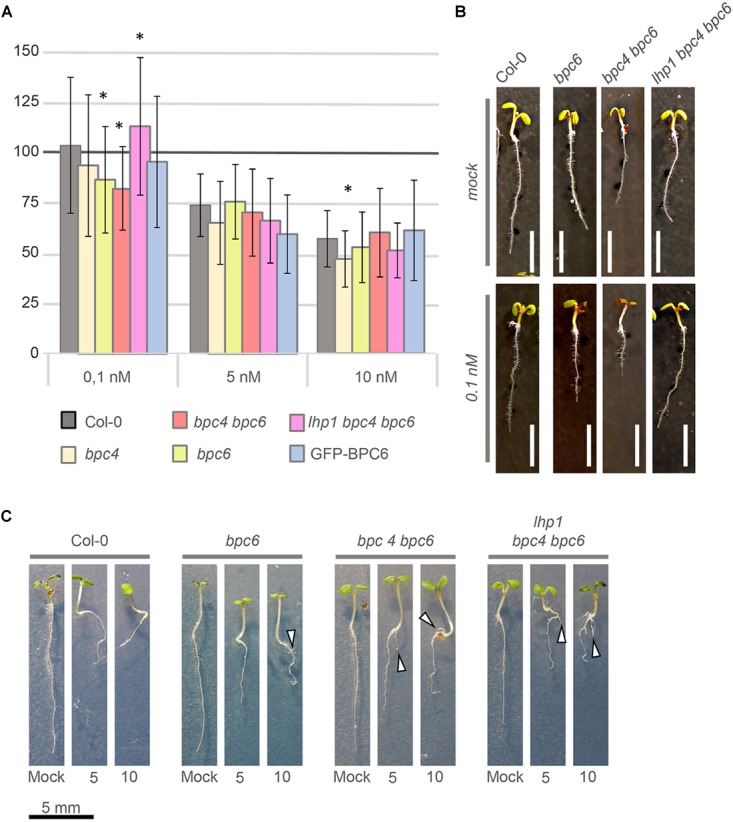
Brassinosteroid sensitivity assay with *bpc*- mutant plants. **(A)** Relative root-growth of 6-day-old seedlings treated with 0.1, 5, or 10 nM brassinolide. Control and treated media were supplemented with 5 nM DMSO. For visualization, total root-length was calculated relative to the control for each genotype. The histogram bars show relative root length normalized to the wildtype Col-0 control (100%). Error bars represent standard deviation. White scale bar represents 4 mm. **(B)** Representative phenotype of selected individuals that showed a significant difference in relative root length at 0.1 nM brassinolide. **(C)** Side root initiation (arrow head) in 8-day-old plants on media supplemented with 5 nM or 10 nM brassinolide. Scale bar for all seedlings is given below the display.

Our findings indicate a direct involvement of BPC6 in the regulation of the entire brassinosteroid signaling pathway. In contrast, a negligible portion of our BPC6 target genes was differentially expressed in the mutants with a compromised *BPC6* function (Figure 9). Therefore, we tested the physiological responses of *bpc4, bpc6, bpc4 bpc6, lhp1 bpc4 bpc6* and p*BPC6*::*GFP-BPC6* plants in the well-established brassinosteroid root sensitivity assay (Perraki et al., 2018 and Figure 12A): Significantly shorter roots were found in *bpc6* and *bpc4 bpc6* mutants at the lowest brassinolide concentration, while *lhp1 bpc4 bpc6* roots were significantly longer than expected (Figure 12A,B). Interestingly, none of the mutants impaired in *BPC6* function displayed significant alterations in root length at higher brassinolide concentrations (Figure 12A). In 8-day-old seedlings, we observed side root initiation in about 80% of the *bpc6, bpc4 bpc6* and in *lhp1 bpc4 bpc6* mutants at higher brassinolide concentrations, which was almost absent in the wildtype plants (Figure 12C). Although this data does not provide a mechanistic clue on the physiological role of BPC6 at the brassinosteroid signaling genes *in vivo*, we can show that mutants impaired in *BPC6* function show altered responses to brassinosteroids.

## Discussion

The data presented here demonstrated that a GAGA/TCTC tetranucleotide motif is required and sufficient for BBR/BPC binding. The possibility that an even shorter binding consensus represents the minimal requirement for BBR/BPC binding is low, because GAG or GAC motifs were already contained in several non-binding oligonucleotide probes (Santi et al., 2003; Meister et al., 2004; Kooiker et al., 2005; Brand et al., 2010; Fischer et al., 2016). Nevertheless, longer GA/TC-repeats possibly allow the binding of more BBR/BPC proteins than expected. This might be permitted by the three-dimensional topology of the DNA, the flexibility of the double helix and, conversely, by sterical hindrance or competition at shorter GA/TC-repeats. A previous study demonstrated that BPC1 has the capacity to induce conformational changes at a large fragment of the *STK* promotor, which contained several longer GA/TC-repeats (Kooiker et al., 2005). It was demonstrated that this BBR/BPC protein was able to bend DNA, which did not depend on isolated, single GAGA-motifs, and indicated that cooperative binding at multiple binding sites might be necessary (Kooiker et al., 2005). One can imagine that a surplus in binding of BBR/BPC proteins to a limited number of GAGA-motifs might induce such conformational changes at longer GA/TC-repeats and explain the higher signal intensities with the BKn3 probe (Figure 6C,D).

Our DPI-ELISA experiment disclosed for the first time, that the motif WAR/KHGTN within the BPC domain is important for GAGA motif recognition. As proposed earlier, the arrangement of the WAR/KHGTN consensus in a putative BPC-dimer will preferentially contact two adjacent GAGA-motifs of the same strand (Wanke et al., 2011; Theune et al., 2017). Consequently, the binding of multiple BBR/BPC-dimers to neighboring GAGA-motifs is consistent with the longer GA/TC-consensi identified from *in vivo* binding studies (Xiao et al., 2017; Shanks et al., 2018). The position of two WAR/KHGTN in parallel oriented dimers will put force onto the DNA during the binding process, which might also contribute to the DNA-bending observed in previous studies with BPC1 (Kooiker et al., 2005). In addition, the highly conserved TIR/K motif terminates almost all BBR/BPC proteins. In our experiment, the removal of these three amino acids reduced the binding to GAGA-motifs already. Two TIR/K motifs in a parallel oriented BBR/BPC dimer might support the WAR/KHGTN in binding to the GAGA-consensus or function as two bearings in the process of DNA-bending.

Previous data demonstrated that regions at the N-terminus are also influencing the binding of BBR/BPC proteins to DNA. In yeast one-hybrid analyses, a fragment of BPC2 that consists only of the highly conserved BPC-domain was unable to induce reporter gene activity, which can be explained by limited or no binding to DNA (Meister et al., 2004). Consistently, a BPC-domain fragment of BPC1 was unable to bind the K4 oligonucleotide *in vitro* (Theune et al., 2017). These data indicate an important function within the N-terminus of group I proteins, that influences DNA-binding of the BPC-domain. Similar observations have been made for other DNA-binding proteins. For example, it was shown that a single amino acid exchange in the catalytic domain of a nuclear beta-amylase transcription factor influences the function of the DNA-binding domain, which is located at the *N*-terminus of the proteins hundreds of amino acids away from mutated site (Soyk et al., 2014).

The WAR/KHGTN motif in BBR/BPC proteins is reminiscent in length and amino acid composition of the conserved WRKYGQK consensus of WRKY proteins (Brand et al., 2013a; Theune et al., 2017). In WRKY proteins, the conserved residues of the WRKYGQK protrude into the major groove of the DNA and make contact to either side of the DNA strands (Brand et al., 2013a). We propose a similar binding mechanism for the WAR/KHGTN motif in BBR/BPC, where some amino acids contact the GA dinucleotide on the one strand and, conversely, the TC dinucleotide on the reverse strand of the DNA.

The reanalysis of the GFP-BPC6 target genes uncovered a significant overlap with brassinosteroid signaling components. An enrichment for GAGA/TCTC motifs close to the region, which was targeted by GFP-BPC6 *in vivo*, is in full agreement with the preference to bind to GAGA-tetranucleotides. It is intriguing that the very same motif occurs in the promoters of co-receptor *BAK1* and the homeotic gene *KNAT1/BP* and constitutes an *in vivo* target of BPC6 in both genes. Targeted ChIP analyses demonstrated that group I protein BPC1 also binds to this motif *in vivo* (Simonini and Kater, 2014), which was the reason that we included the GAGAGATGAGAGA motif in our *in vitro* studies (Figure 6). The observation of the same motif in *BAK1* and *KNAT1/BP* suggest a similar effect by BBR/BPC proteins on both genes. Although the overall expression was not significantly altered in mutant plants, the expression of *KNAT1/BP* in the inflorescence meristem of *bpc1 bpc2 bpc3* triple-mutants was induced (Simonini and Kater, 2014).

It was proposed that the effect of BBR/BPC proteins on the meristem size is a result of their influence on homeotic genes such as WUS or Knotted-like genes (Santi et al., 2003; Simonini and Kater, 2014). Several homeotic genes are well-known integrators of phytohormone signaling. BBR/BPC proteins of different groups were shown to bind to Cytokinin responsive genes *in vivo* (Simonini and Kater, 2014; Shanks et al., 2018). We reanalyzed the GFP-BPC6 ChIP-data and uncovered a link with brassinosteroid signaling components. This function for BPC6 in brassinosteroid signaling was consolidated in our physiological experiments. Our data suggest that BPC6 might be involved in the concerted activation or repression of the entire pathway. Interestingly, BPC6 targets also the promotor of the phytosulfikine receptor PSKR1, that also interacts with the promiscuous brassinosteroid signaling component BAK1 or the H^+^-ATPases AHA1 and AHA2 (Ladwig et al., 2015). Moreover, the promotors of CLV1 or TIR1 are also contained in the target gene list of BPC6, which all might contribute to the developmental changes observed in higher order *BBR/BPC* mutants (Monfared et al., 2011; Simonini and Kater, 2014; Hecker et al., 2015). So far, a role for BBR/BPC proteins in phytohormone signaling was shown for brassinolide (this study), cytokinin and ethylene (Monfared et al., 2011; Simonini and Kater, 2014; Shanks et al., 2018).

Our phylogenetic analyses on the BPC domains and the full-length proteins suggest the separation of the known groups of BBR/BPC proteins into further subgroups. In addition, we propose a group IV that contains only liverworts, mosses, ferns and gymnosperm and that is basal to all other BBR/BPC groups. Cryptogams are missing in groups I, II and III, which are dominated by angiosperm representatives. Gymnosperms are the only seed plants that contain basal group IV as well as groups I amd II BBR/BPC members. We also provided evidence that group III is of polyphyletic origin and is supplemented by truncated group I or II BBR/BPC proteins, which presumably become pseudogenes in the near future. Interestingly, no BBR/BPC proteins were uncovered in any other phylum besides land plants. The most ancient representatives of the BBR/BPC family can be found in the genome of the liverwort *Marchantia*.

Investigation of homeotic genes, for example LFY, AP2 or MADS transcription factor genes are well known for their importance in flower development and constitute elements that act downstream of or in concert with group I and group II BBR/BPC proteins (Liu et al., 2009; Winter et al., 2011; Hecker et al., 2015; Silva et al., 2015; Wang et al., 2016; Xiao et al., 2017; Shanks et al., 2018). These gene families display a huge diversification and expansion during land plant evolution and, especially, within the rosids and asterids, which can be explained by whole genome duplications in the early angiosperms (Scutt and Vandenbussche, 2014; Silva et al., 2015; Wang et al., 2016). Although BBR/BPC proteins also diversified, the number of functional paralogous proteins is surprisingly low in most clades compared to LFY, AP2 or MADS proteins. For example, there are no true paralogs in Arabidopsis group II proteins: As BPC4 and BPC6 reside in different subgroups, the sole remaining paralog to BPC4 is the pseudgene BPC5 (Monfared et al., 2011; Wanke et al., 2011; Simonini and Kater, 2014). Moreover, some group I specific functions are probably missing from the Arabidopsis lineage, because these proteins lack certain domains.

One of the central questions in plant evolutionary developmental biology is how the reproductive flower structure evolved (Scutt and Vandenbussche, 2014). There is a tight linkage between BBR/BPC diversification in the land plant lineage and the complexity of flower and seed plant evolution (Lang et al., 2010): One, we provide phylogenetic evidence that the common ancestor of group I and II proteins was a seed plant already, and diversification occurred mainly in the asterids, where other organizational levels of flowers evolved (Scutt and Vandenbussche, 2014). Two, many floral homeotic genes are validated downstream targets of BBR/BPC proteins *in vivo* and are controlled in their expression mainly through repressive mechanism (Santi et al., 2003; Berger et al., 2011; Monfared et al., 2011; Simonini et al., 2012; Simonini and Kater, 2014; Hecker et al., 2015; Xiao et al., 2017; Shanks et al., 2018). Three, BBR/BPC proteins are involved in phytohormone signaling, possibly by targeting the promoter regions of genes involved in signal transduction (Monfared et al., 2011; Simonini and Kater, 2014; Shanks et al., 2018). Here we show that BPC6 is an upstream element of many genes involved in brassinosteroid signaling. It is noteworthy, that the evolution of brassinosteroid phytohormones and, especially, of brassinolide is tightly connected to the evolution of reproductive organs more than any other phytohormone (Hartwig et al., 2011; Belkhadir and Jaillais, 2015; Wang et al., 2015; Yokota et al., 2017). The targeting of an almost entire signaling pathway by one transcriptional regulator implies a requirement for simultaneous activation or repression. The molecular function of BPC6 in this context remains unclear and requires further investigation in the future.

## Materials and Methods

### Phylogenetic Analysis and Protein Domain Sequence Alignment

BBR/BPC protein data were predominantly obtained for those species in which genome sequence was available. All sequences were retrieved from the NCBI database^1^. Searches for basal sequences, especially from lineages outside the land plants, was performed by using tBLASTn against nucleotide databases (nr, est, htgs, gss) with various protein sequences as input and with organism identifiers for Characeae (taxid:3146), Chlorophyta (taxid:3041), Phaeophyceae (taxid:2870) and Rhodophyceae (taxid:2763) at the NCBI^1^. Multiple protein sequence alignments were performed using M-Coffee (packages: PCMA, Mafft, Muscle, T-Coffee, ProbCons) (Wallace et al., 2006; Di Tommaso et al., 2011). Phylogenetic analyses were performed using phylogeny.fr (Dereeper et al., 2008). Neighbor-Joining trees (BioNJ) (Gascuel, 1997) were constructed based on Jones-Taylor-Thornton matrices (Jones et al., 1992). To asses support for each node, 1000 bootstrap samples were generated (Felsenstein, 1985). TreeDyn (Chevenet et al., 2006) and Dendroscope (Huson et al., 2007) were used for visualization and editing of the trees. Branches with <20% bootstrap support were collapsed.

Conserved protein motifs outside the highly conserved DNA-binding domain were analyzed by using the applications MEME or GLAM in the MEME suit^2^ tool collection. Due to the diversity at the *N*-terminus of the proteins, motif discovery was performed for each group or subgroup independently. Any number of repetitions was accepted. The MEME data mining was performed as follows: Number of motifs 3 to 10; minimum width 10; maximum width 110. Motif consensi that were present in several groups or subgroups were subsequently unified. GLAM was used for the identification of motif rearrangements, gapped or truncated motifs. WebLogo^3^ was used to generate the sequence logos of conserved protein motifs (Crooks et al., 2004).

### Protein Expression and Detection by Western Blotting

The open reading frame of BASIC PENTACYSTEINE 1 (BPC1; AT2G01930) was amplified by PCR from a cDNA-library from *Arabidopsis thaliana* flowers without a stop codon for subsequent cloning into pENTR/D-TOPO (Invitrogen) (Theune et al., 2017). Truncations of the protein were generated by PCR. After sequencing, the specific insert was recombined *via* Gateway (Invitrogen) LR-reaction into the appropriate pET32b-GW destination vector (Ciolkowski et al., 2008; Brand et al., 2010). This vector provides translational fusion of a 6×His-epitope to the *N*-terminus of the proteins of interest (Ciolkowski et al., 2008). Expression of recombinant BPC1^6×His^ and derivatives was performed in *Escherichia coli* BL21/RIL. Expression and extraction of recombinant GFP-BPC6 (BPC6; At5g42520) has previously been demonstrated (Hecker et al., 2015). Protein extraction was performed according to the DPI-ELISA protocol (Brand et al., 2010). Protein extracts from untransformed *E. coli* BL21/RIL cells were used as a negative controls.

For protein detection, the crude extracts were first separated by SDS-PAGE in a Mini-PROTEAN Tetra Vertical Electrophoresis Cell (BioRad): Standard cast 10% SDS Mini gels (BioRad) were prepared according to the manufacturer’s description (Theune et al., 2017). Stacking gels contained 0.125 M Tris (pH 6.8), 0.1% (w/v) SDS, 0.04% (v/v) TEMED, and 0.4% (w/v) APS. Separating gels contained 0.375 M Tris (pH 8.8), 0.1% (w/v) SDS, 0.025% (v/v) TEMED, and 0.125% (w/v) APS. Native protein extracts were mixed with a Laemmli sample buffer [62.5 mM Tris (pH 6.8), 4% SDS, 20% glycerol, 0.01% bromophenol blue, 5% β-mercaptoethanol] and heated for 10 min at 95°C prior to loading. Electrophoresis was performed at 100 V in Tris-Glycine SDS running buffer [250 mM Tris, 1.92 M Glycine, 1% SDS, pH 8.3] (Sambrook and Russell, 2001). Spectra Multicolor Broad Range Protein Ladder (Fermentas) was used for mass estimation (kDa). Protein gels were blotted using a Mini Trans-Blot Cell (BioRad) and PVDF membranes (Millipore). Membranes were blocked with 5% non-fat dry milk (Roth) in TBS-T. Mouse anti-His primary antibody (Qiagen)(1/2000) and alkaline phosphatase (AP)-conjugated rabbit anti-mouse (Qiagen)(1/5000) were used with NBT/BCIP solution (Roche) for subsequent chromogenic detection (Theune et al., 2017).

### DPI-ELISA and qDPI-ELISA

DNA binding properties for BPC1^6×His^ and derivatives were analyzed using the DPI-ELISA method (Brand et al., 2010, 2013b; Theune et al., 2017). Binding of GFP-BPC6 to different double-stranded oligonucleotides was studied by the qDPI-ELISA protocol (Fischer et al., 2016). Per microplate well, 3 μg total protein in buffered solution and 2 pmol biotinylated double-stranded oligonucleotides were used. Biotinylated (sense) and non-biotinylated (antisense) single-stranded oligonucleotides were ordered from Biomers (Germany) and hybridized *in vitro* (Fischer et al., 2016). The oligonucleotide sequences of the sense strand are shown in Figure 6A. Each DPI-ELISA or qDPI-ELISA experiment was repeated at least twice on different ELISA-plates, with different protein extracts and with two technical replicates per plate. Total protein from two independent extractions was used. A Tecan Safire plate reader was used for photometric and fluorometric detection. Statistical analysis was performed in Microsoft Excel. To test for significant differences between the mean fluorescence values of the qDPI-ELISA, a one-way ANOVA [*F*(8,18) = 96.344, *p* = 0.001] was performed with the Analysis of Variance (ANOVA) Calculator by D.S. Soper^4^.

### Protein Folding and Structure Prediction

The PDB files of the monomeric or dimeric Arabidopsis BPC1 model structures (Theune et al., 2017) were imported into PyMOL^5^, to derive a 3D structure model. The dimer structure was also imported in Foldit Standalone (Khatib et al., 2011a,b) and modified using the mutation functions to generate the BPC6-binding domain. The function Minimize Sidechains was used first and the Minimize Backbone later to realize the model. Two loops had to be further changed by using the Rebuild tool on a short region around His258. The final model was exported to a PDB file and visualized in PyMol.

### Metadata Analysis

The raw GFP-BPC6 and control GFP ChIP-sequence data (Shanks et al., 2018) were processed using the Illumina sequence data analysis pipeline GAPipeline1.3.2. Subsequently, Bowtie (Langmead et al., 2009) was used to map the reads to the *Arabidopsis* genome (TAIR10) (Lamesch et al., 2012). Only perfectly mapped reads were retained for analysis (FDR < 0.05). Peak calling was performed with MASC (Zhang et al., 2008) independently for each replicate combination. The median of the fold-increase between the GFP-BPC6 replicates and the GFP control replicates was computed for all 4 replicate combinations independently by using the tool package in the Integrated Genome Browser (IGB) (Freese et al., 2016). Bedgraphs were merged and visualized either with the Integrated Genome Viewer (IGV) (Robinson et al., 2011; Thorvaldsdottir et al., 2013) or the Integrated Genome Browser (IGB) (Freese et al., 2016).

Motif discovery and WebLogo illustration in the promoters of brassinosteroid signaling genes was performed with the MEME suit^2^ (Bailey et al., 2009), with the following settings modified from the default: Maximum number of motifs 5, motif *E*-value threshold no limit, minimum motif width 4, maximum motif width 7, minimum sites per motif 2 and any number of repetitions (anr). The heat map and GAGA/TCTC motif distribution analyses were performed with PIL and matplotlib.pyplot routines in Python3 in a Spyder3 environment^6^.

Metadata comparison with expression information from different microarray experiments was performed as described before (Wenke et al., 2012; Hahn et al., 2013). Selected microarray experiments of the AtGenExpress hormone and chemical treatment data set were used in this study (Goda et al., 2008): Affymetrix CEL files ME00334 (response to 1-aminocyclopropane-1-carboxylic acid), ME00335 (response to brassinolide), ME00336 (response to indole-3-acetic acid), ME00343 (response to gibberellic acid), ME00364,(response to salicylic acid) were retrieved from The Arabidopsis Information Resource^7^. Normalization and evaluation of the microarray data were carried out as was described before (Kilian et al., 2007). The list of brassinosteroid signaling components was hand edited by manual literature searches. Gene list overlaps and Venn diagrams were compiled in Venny2.1^8^, which is based on the Venn Diagram Generator by C. Seidel^9^. Significant overlaps between datasets were calculated using the hypergeometric distribution function.

### Plant Material and Brassinosteroid-Sensitivity Assay

All plants used here were in the *Arabidopsis thaliana* accession Col-0 background. The following genotypes have been previously reported: *bpc4* (Monfared et al., 2011), *bpc6* (Monfared et al., 2011), *bpc4 bpc6* (Monfared et al., 2011; Hecker et al., 2015), *lhp1 bpc4 bpc6* (Hecker et al., 2015) and p*BPC6*::*GFP-BPC6* (Shanks et al., 2018). Seeds were surface-sterilized and grown in square Petri dishes. Individual seeds were placed in line on solid media (1/2 MS, 1% sucrose and 0.8% phytoagar). For root growth assays, media was supplemented with a final concentration of 5nM DMSO (Mock) or either 0.1, 5 or 10 nM Brassinolide. The plates with seeds were stratified at 4°C for 2 days and then placed vertically in a growth chamber with continuous light for 6 to 10 days. Each day, the position of the root tip was marked. Total root length was measured 6 days post germination with a ruler to the full millimeter. Pictures of the plates were taken at the final day of the experiment, before the total root lengths were measured. Pictures of the root branching phenotype were taken at 8 days post germination. To rule out genotypic differences between the plants, the root lengths of each genotype were normalized to their respective control condition, before comparing to the Col-0 roots. The experiments were repeated twice independently in two replicates each: Col-0 [*n* = 33–40], bpc4 [*n* = 18–22], bpc6 [*n* = 25–27], bpc4 bpc6 [*n* = 33–40], lhp1 bpc4 bpc6 [*n* = 33–40] and p*BPC6*::*GFP-BPC6* [*n* = 33–40]. Statistical analysis was performed by student’s *T*-test on raw and normalized values.

## Author Contributions

MT, UB, LB, FL, and DW contributed conception and design of the study and wrote sections of the manuscript. MT computed the predicted model structures and analyzed the sequence data together with FL and DW. UB and DW compiled and evaluated the phylogenetic trees. LB and DW analyzed the protein and gene structures. DW performed the DPI-ELISA and qDPI-ELISA and wrote the first draft of the manuscript. FL and DW performed and analyzed the physiological experiments with plants. MT and DW performed statistical analysis. All authors contributed to manuscript revision, read and approved the submitted version.

## Conflict of Interest Statement

The authors declare that the research was conducted in the absence of any commercial or financial relationships that could be construed as a potential conflict of interest.

## Footnotes

1 http://www.ncbi.nlm.nih.gov

2 http://meme-suite.org/

3 http://weblogo.berkeley.edu/logo.cgi

4 http://www.danielsoper.com/statcalc

5 http://www.pymol.org

6 https://www.spyder-ide.org/

7 http://www.arabidopsis.org

8 http://bioinfogp.cnb.csic.es/tools/venny/

9 http://www.pangloss.com

## Supplementary Material

IMAGE S1 Structural models for the BPC domains of BPC1 and BPC6. Derived model structures for the BPC domains of BPC1 (top; green) and BPC6 (bottom; red). Conserved amino acid residues are highlighted in gray color. Conserved Cysteines are indicated in yellow. The unique Histidin in BPC6 is indicated in blue color.

TABLE S1 List of 83 BPC-domain sequences used for the cladogram.

TABLE S2 List of 68 full-length BBR/BPC protein sequences used for the phylogram.

TABLE S3 Fluorescence of recombinant GFP-BPC6 in the qDPI-ELISA experiment with diverse DNA-probes.

TABLE S4 Regions associated with a gene locus that are significantly enriched in both GFP-BPC6 replicates compared to the GFP-controls.

TABLE S5 List of 48 BPC6 *in vivo* target genes of the brassinosteroid signaling pathway.

TABLE S6 920 genes differentially expressed in *bpc1,2,3,4,6* mutant roots.

TABLE S7 Genes responsive after phytohormone treatment.

TABLE S8 Peak and promotor data of those 47 brassinosteroid signaling genes that are significantly targeted by BPC6 *in vivo*.

DATA SHEET S1 Conserved amino acid residues in 59 BPC domains of different groups and subgroups. Different colors differentiate between the four major BBR/BPC groups. The invariant cysteines are indicated by black background. Other residues that are invariant within each subgroup are highlighted by gray background.

DATA SHEET S2 Conserved protein sequence motifs outside the BPC domain. Overview over 14 conserved peptide motifs that are characteristic for group I to IV BBR/BPC proteins outside their invariant BPC domain.

DATA SHEET S3 Visualization of additional GFP-BPC6 targets in the brassinosteroid signaling pathway. BPC6 binding sites upstream of multiple genes involved in the brassinosteroid response are visualized by using the Integrative Genome Browser. Raw GFP-BPC6 binding data were published before (Shanks et al., 2018). The top bedgraph displays GFP-BPC6 binding data; the second bedgraph shows binding of the negative GFP control. The indicated genes are shown above each panel, with boxes corresponding to exons and bars to intron, with the direction of transcription indicated by small arrows. The location of the sequence targeted by BPC6 and the position of GAGAGA or TCTCTC are shown at the bottom of each panel. The shown data range for each investigated gene is shown in the top left corner.

DATA SHEET S4 Map of GAGA/TCTC motifs in the brassinosteroid signaling genes. Heat map distribution of the GAGA/TCTC in the brassinosteroid signaling genes with gene name and AGI. The map was centered to the highest binding peak. Red, TCTC; Blue, GAGA.
