# Supplementary figures and images for: Phylogenetic Analyses and GAGA-Motif Binding Studies of BBR/BPC Proteins Lend to Clues in GAGA-Motif Recognition and a Regulatory Role in Brassinosteroid Signaling
Source: Front Plant Sci. 2019 Apr 16;10:466. doi: 10.3389/fpls.2019.00466 (PMC6477699; doi:10.3389/fpls.2019.00466)

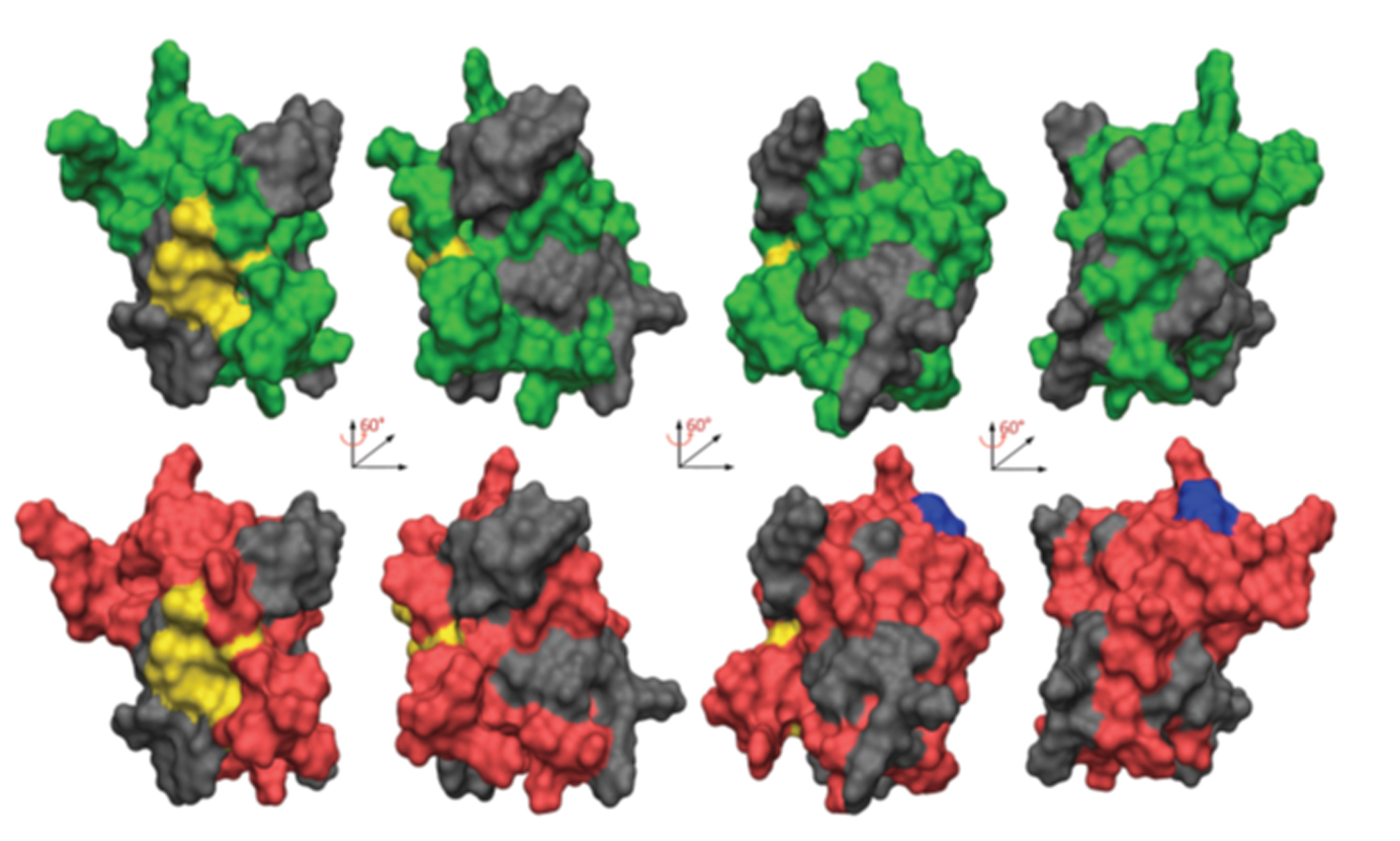

Supplement: IMAGE S1 — Structural models for the BPC domains of BPC1 and BPC6. Derived model structures for the BPC domains of BPC1 (top; green) and BPC6 (bottom; red). Conserved amino acid residues are highlighted in gray color. Conserved Cysteines are indicated in yellow. The unique Histidin in BPC6 is indicated in blue color. [file Image_1.JPEG]

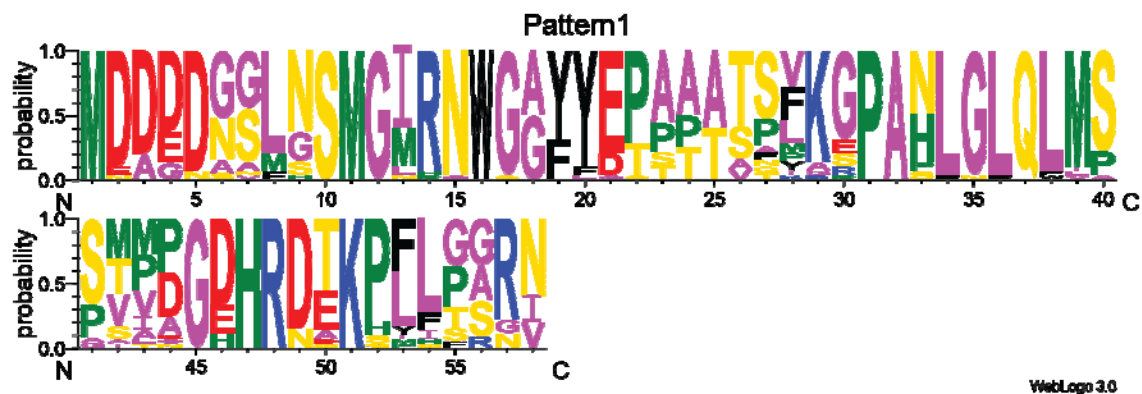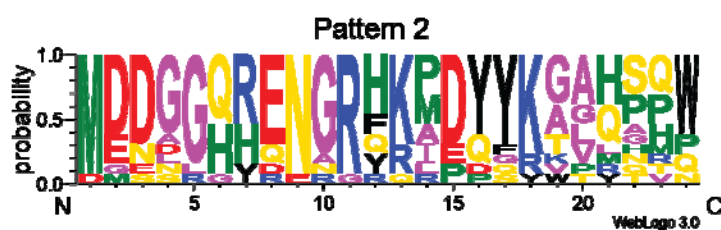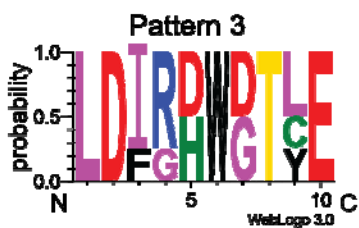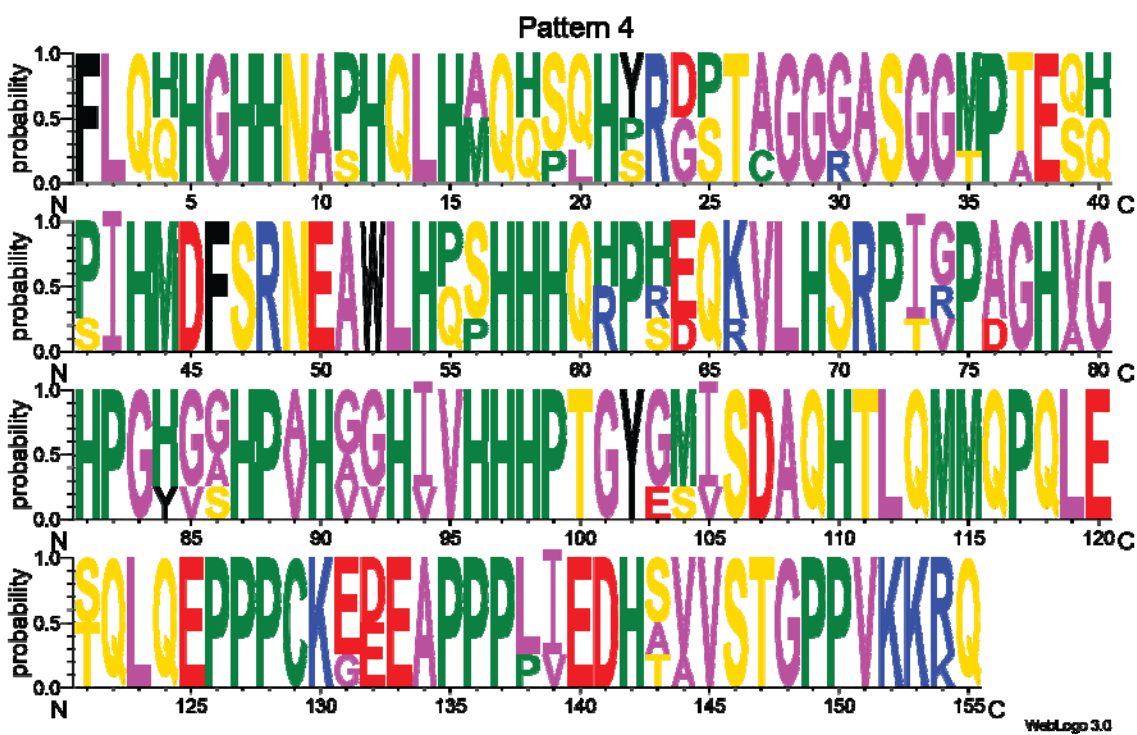

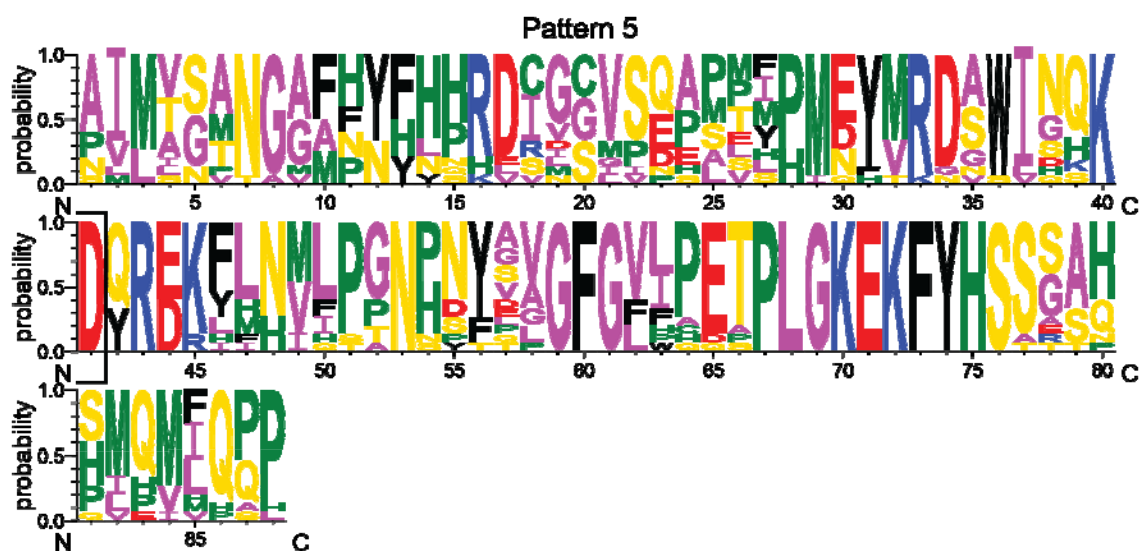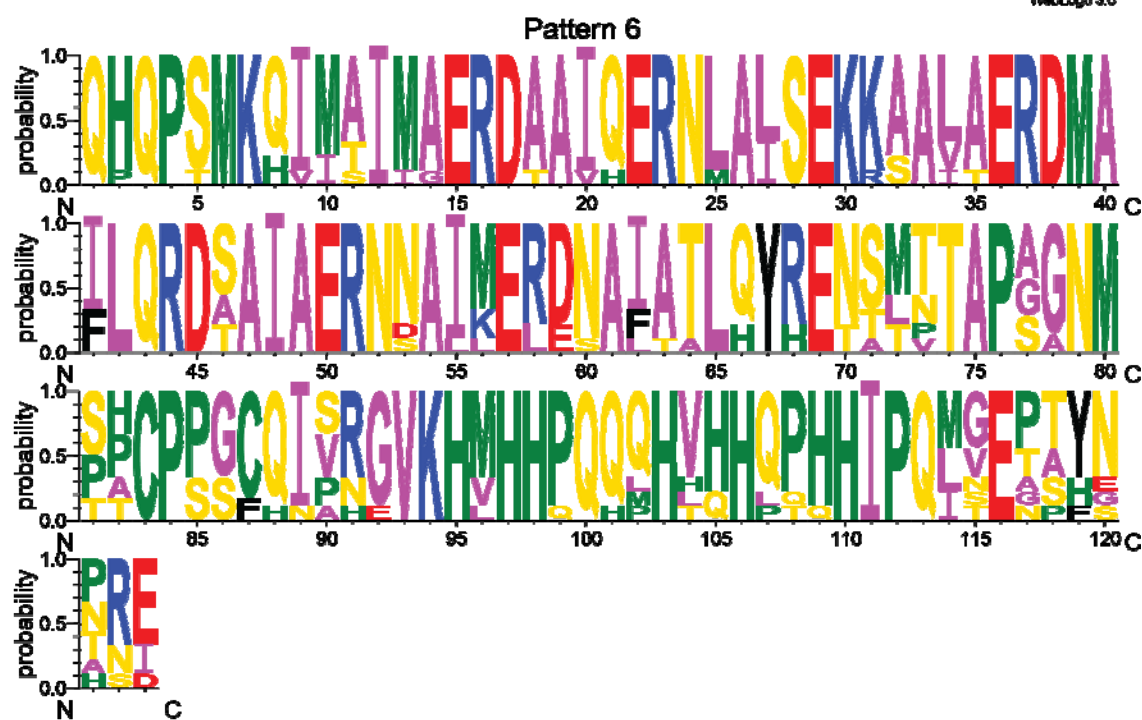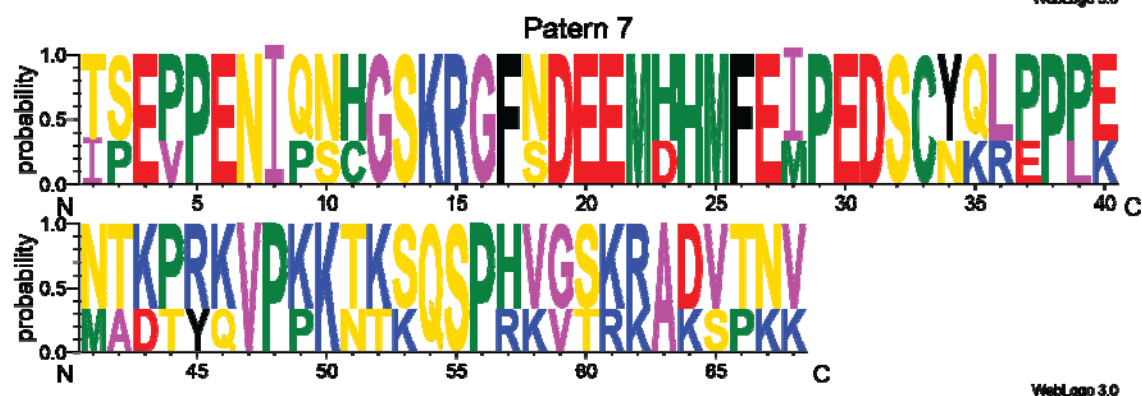

Pattern 8

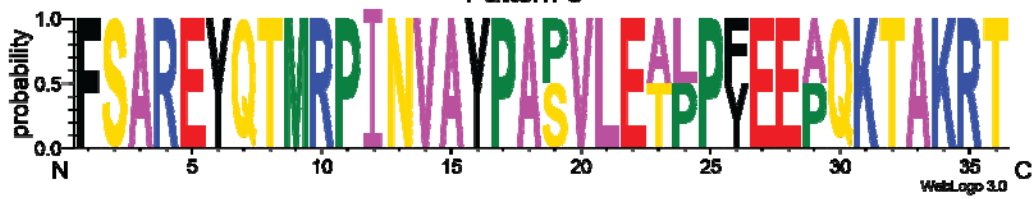

Pattern 9

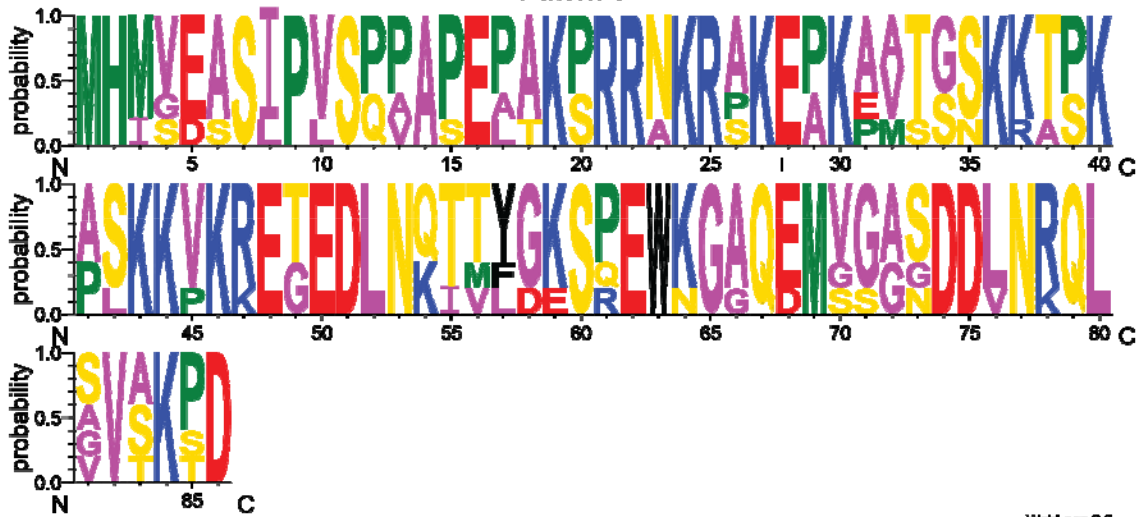

WebLogo 3.0

Pattern 10

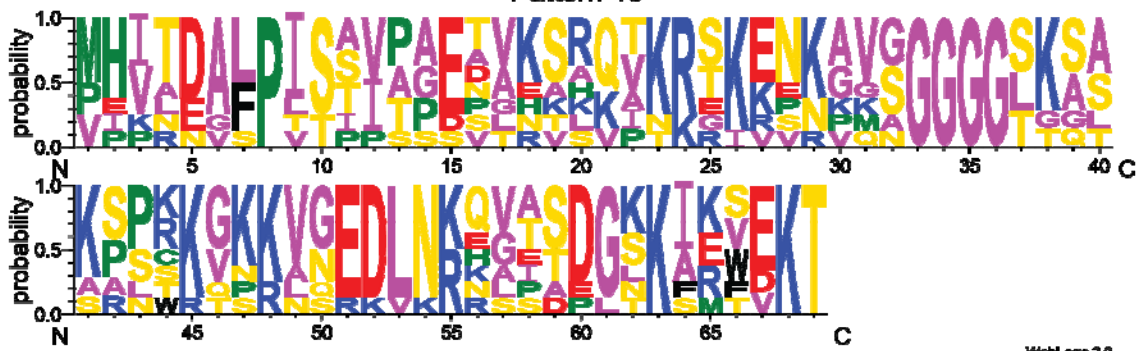

WebLogo 3.0

Pattern 11

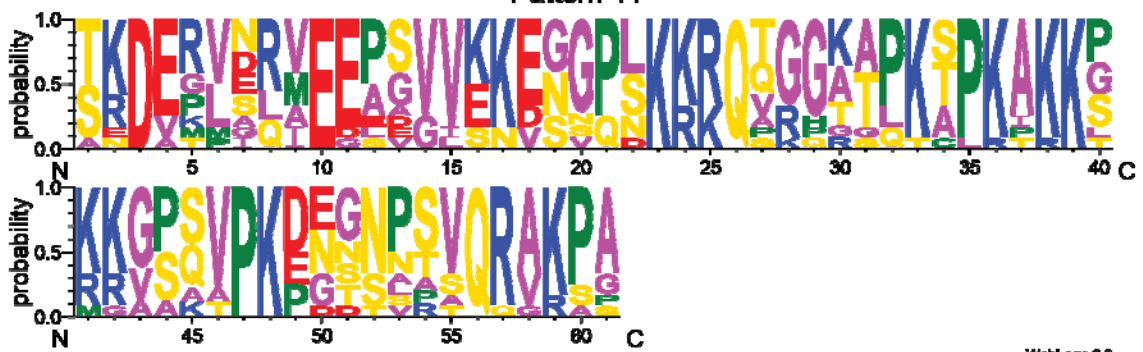

WebLogo 3.0

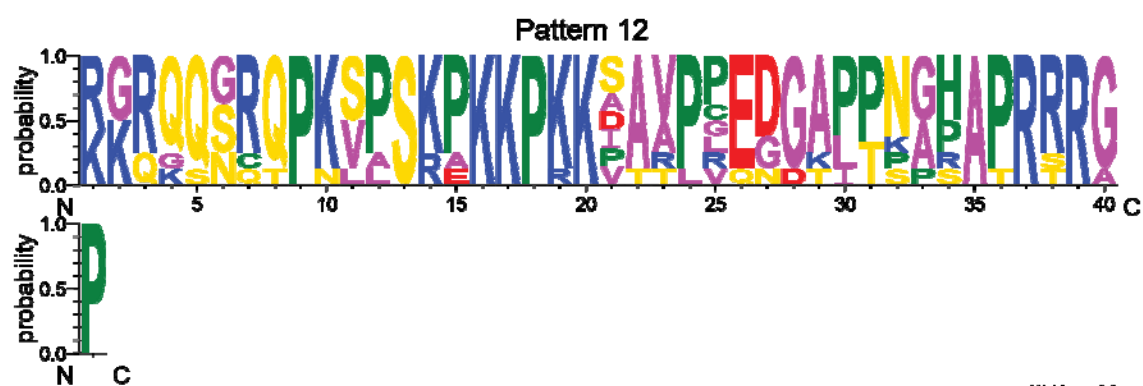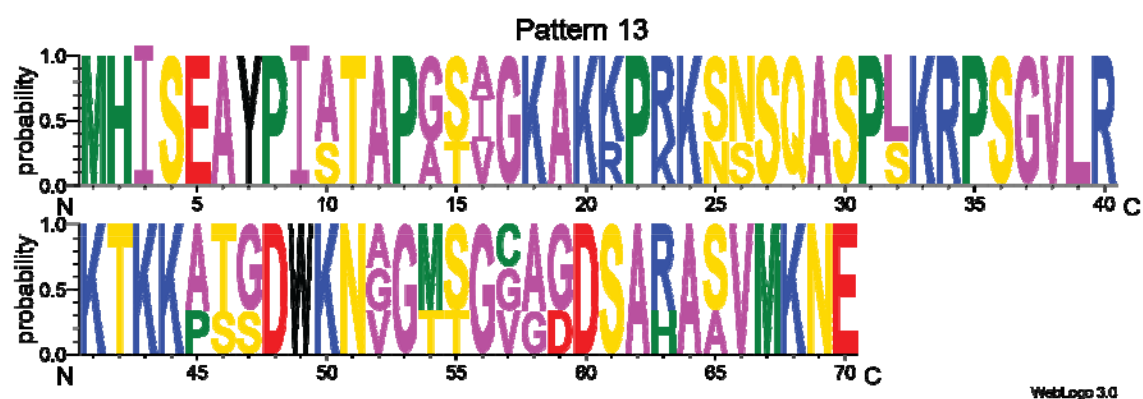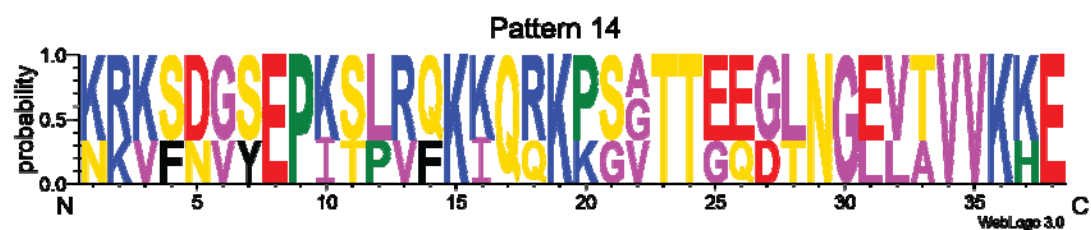

Supplement: DATA SHEET S2 — Conserved protein sequence motifs outside the BPC domain. Overview over 14 conserved peptide motifs that are characteristic for group I to IV BBR/BPC proteins outside their invariant BPC domain. [file Data_Sheet_2.PDF]

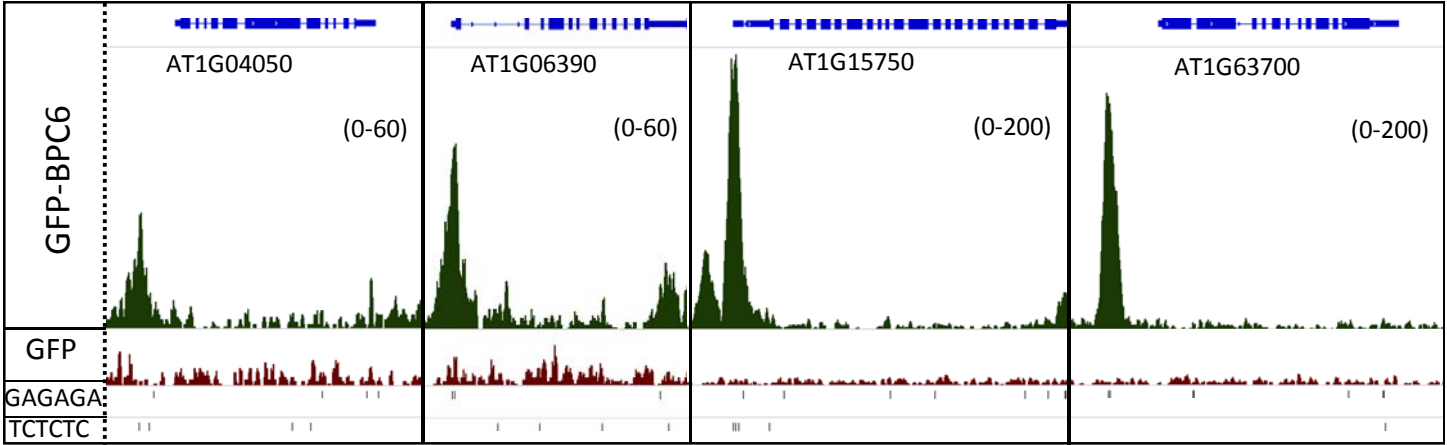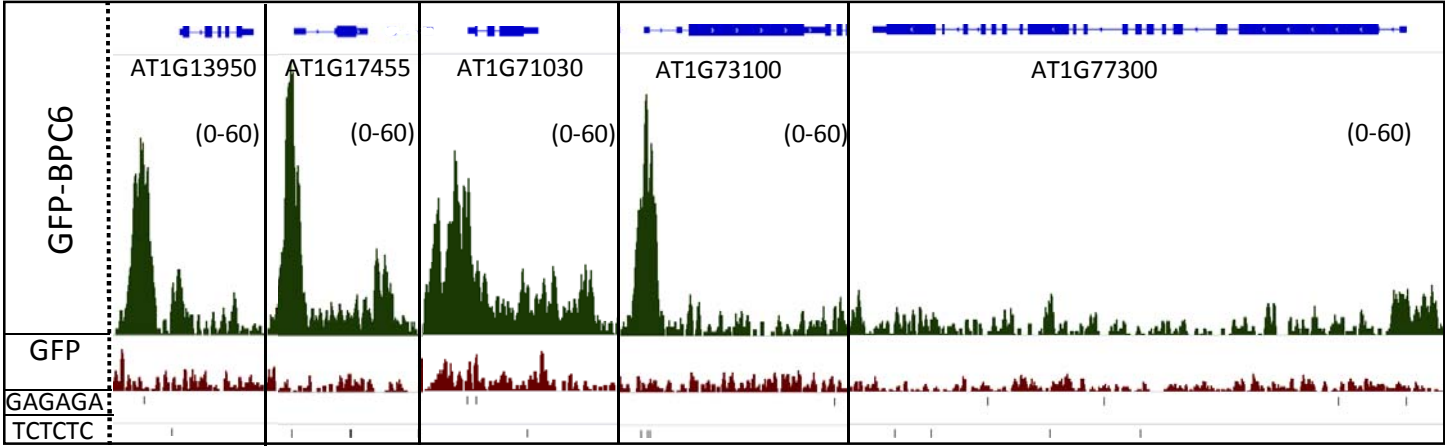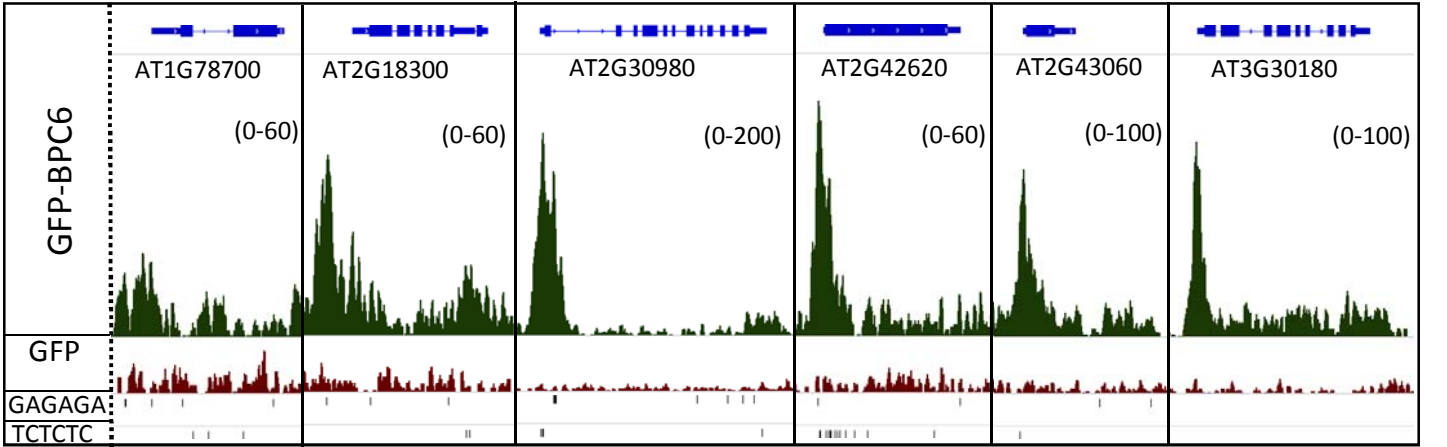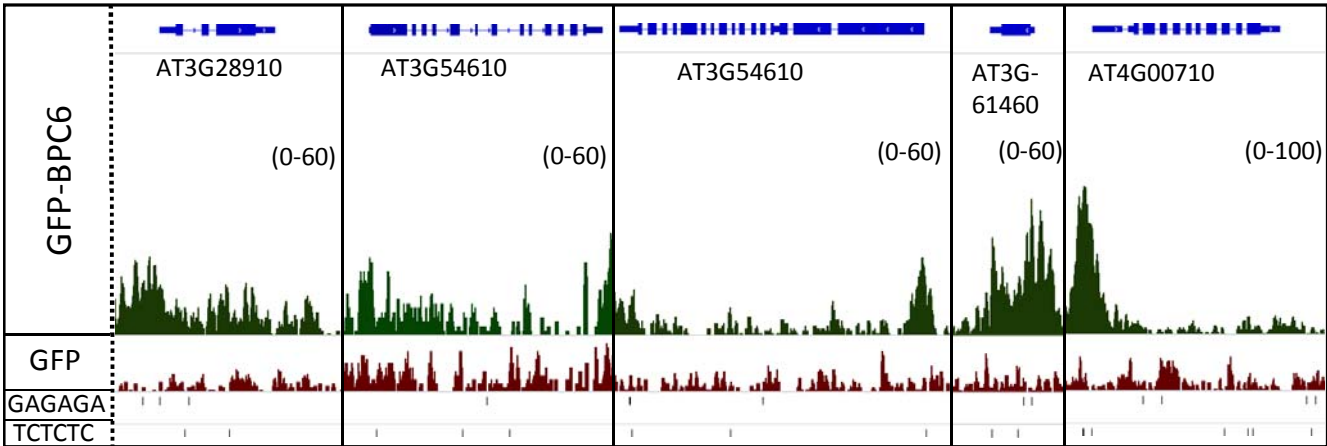

← 5kb →

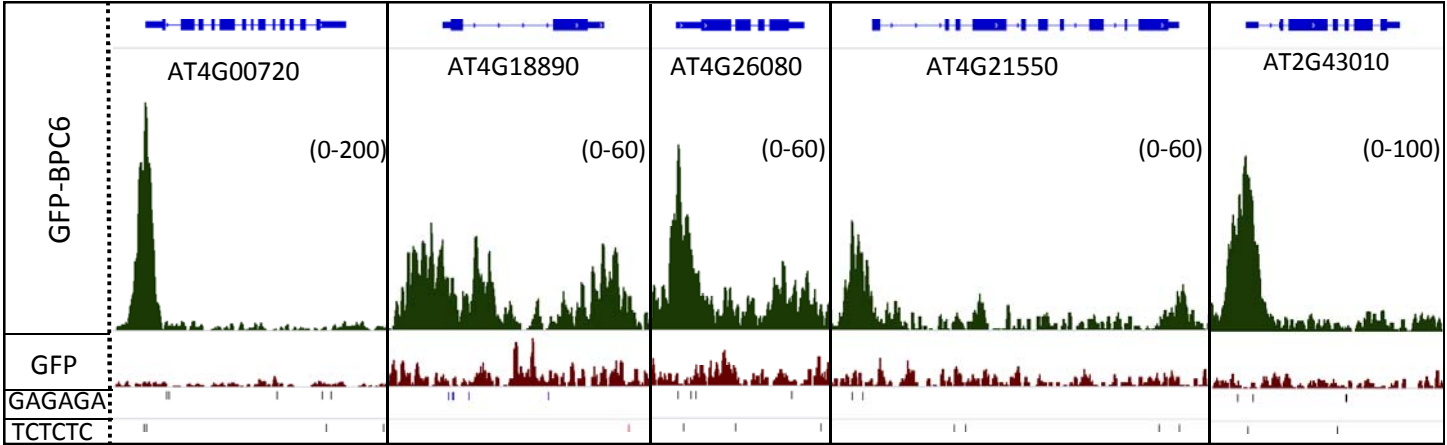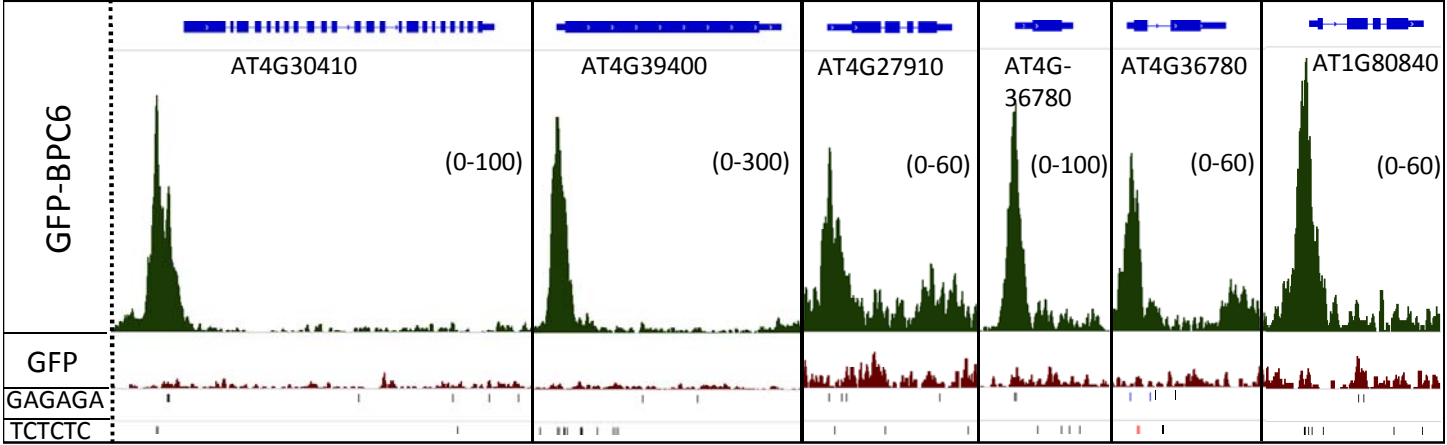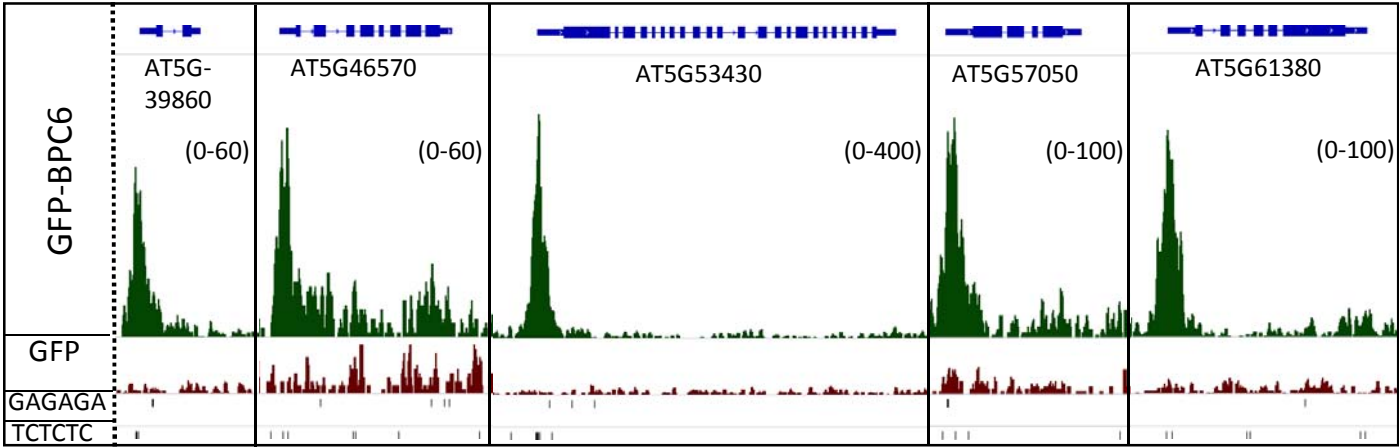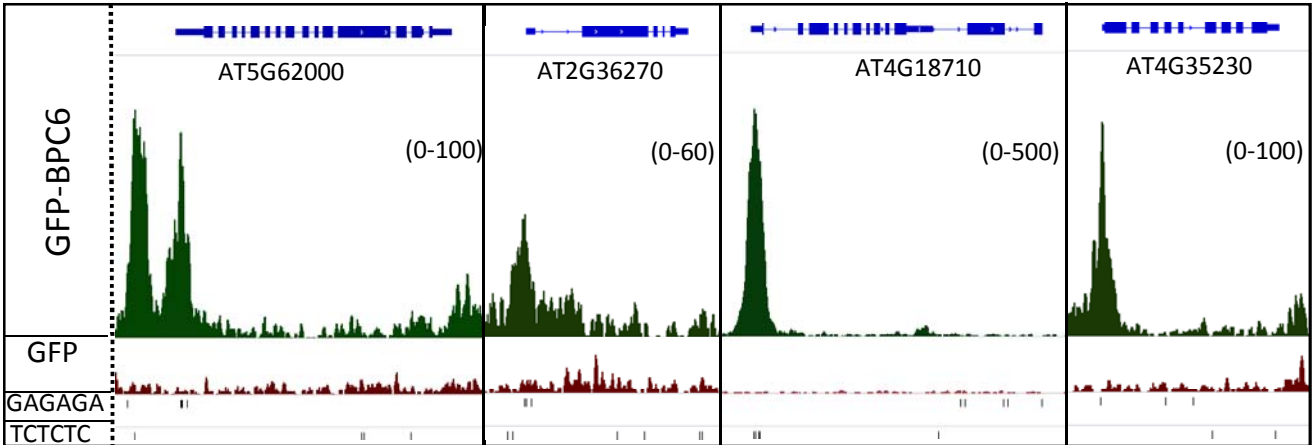

← 5kb →

Supplement: DATA SHEET S3 — Visualization of additional GFP-BPC6 targets in the brassinosteroid signaling pathway. BPC6 binding sites upstream of multiple genes involved in the brassinosteroid response are visualized by using the Integrative Genome Browser. Raw GFP-BPC6 binding data were published before (Shanks et al., 2018). The top bedgraph displays GFP-BPC6 binding data; the second bedgraph shows binding of the negative GFP control. The indicated genes are shown above each panel, with boxes corresponding to exons and bars to intron, with the direction of transcription indicated by small arrows. The location of the sequence targeted by BPC6 and the position of GAGAGA or TCTCTC are shown at the bottom of each panel. The shown data range for each investigated gene is shown in the top left corner. [file Data_Sheet_3.PDF]

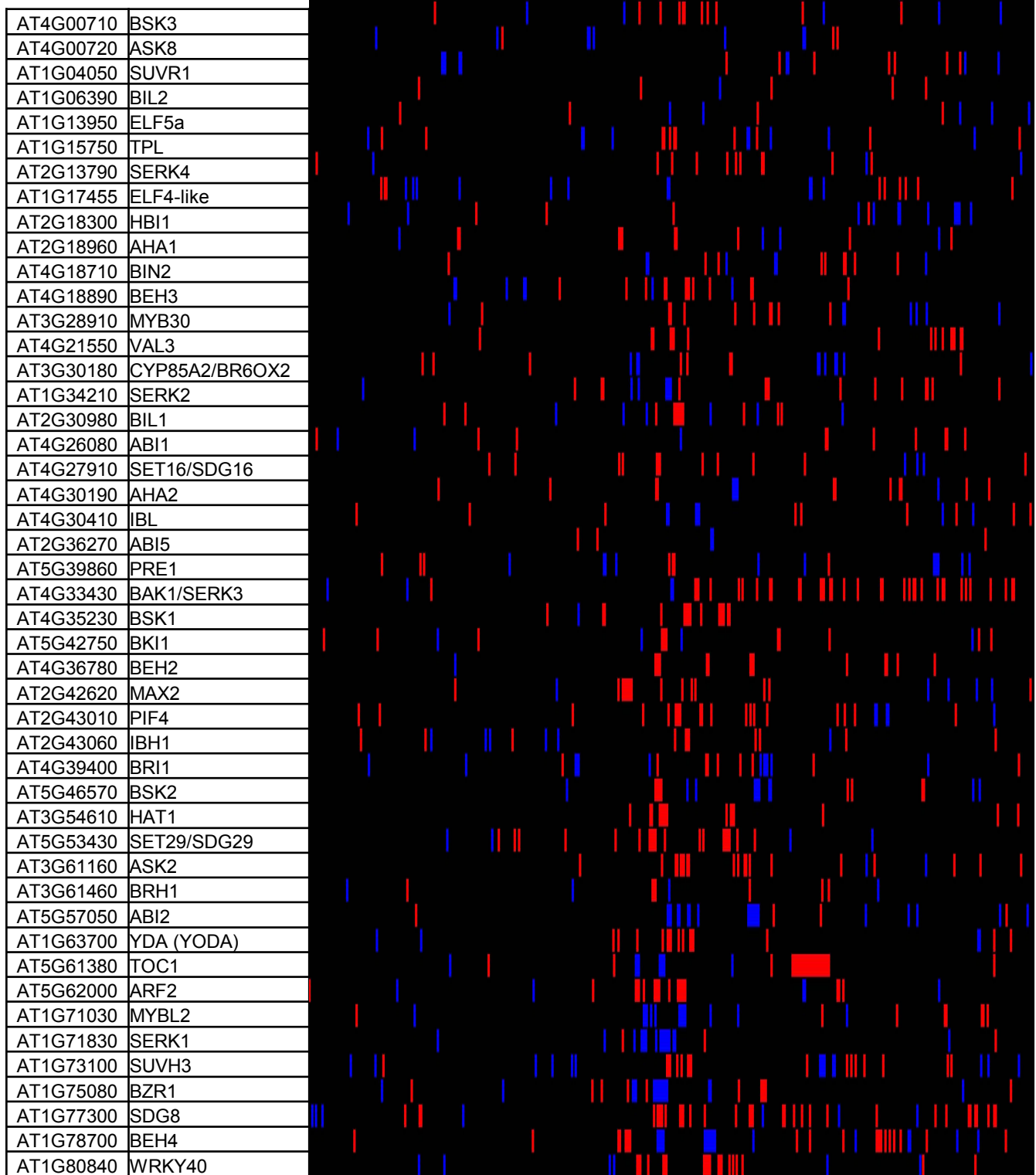

Supplement: DATA SHEET S4 — Map of GAGA/TCTC motifs in the brassinosteroid signaling genes. Heat map distribution of the GAGA/TCTC in the brassinosteroid signaling genes with gene name and AGI. The map was centered to the highest binding peak. Red, TCTC; Blue, GAGA. [file Data_Sheet_4.PDF]
