# Supplementary material for: Phylogenetic Analyses and GAGA-Motif Binding Studies of BBR/BPC Proteins Lend to Clues in GAGA-Motif Recognition and a Regulatory Role in Brassinosteroid Signaling
Source: Front Plant Sci. 2019 Apr 16;10:466. doi: 10.3389/fpls.2019.00466 (PMC6477699; doi:10.3389/fpls.2019.00466)
Supplement: DATA SHEET S1 — Conserved amino acid residues in 59 BPC domains of different groups and subgroups. Different colors differentiate between the four major BBR/BPC groups. The invariant cysteines are indicated by black background. Other residues that are invariant within each subgroup are highlighted by gray background. [file Data_Sheet_1.PDF]

Group I

Group IA

```
GmGAGA-BP      NGIDMDISSIPIPVCSCTGAPQQCYKWGSGGWQSACCTTGMSVYPLPMSTKRRGARIAGRKMSIGAFKKVLEKLAAEG-YNFSNPIDLRTYWAKHGTNKFVTIR
PtBPC8         NGINMDISVIPIPVCSCTGNPQQCYRWGCGGWQSACCTTCISVYPLPMSTKRRGARIAGRKMSLGAFKKVLEKLAGEG-YDFSNPIDLRTHWAKHGTNKFVTIR
PtBPC9         NGINMDISVIPIPVCSCTGNPQQCYRWGCGGWQSACCTTCISVYPLPMSMKRRGARIAGRKMSLGAFKKVLEKLAGEG-YDFSNPIDLRTHWAKHGTNKFVTIR
PtBPC10        NGINMDMSVVPIPVCSCTGNAQQCYRWGCGGWQSACCTTCISMYPLPMSTKRRGARIAGRKMSSGAFKKVLEKLADEG-YDFSNPIDLRTHWAKHGTNKFVTIR
PtBPC11        NGISMDISLFPIPVCSCTGTPQQCYRWGCGGWQSACCTTCISVHPLPMSMKRRGARIAGRKMSLGAFKKVLEKLAGEG-YDFSNAIDLRTHWAKHGTNKFVTIK
VvBBR/BPC2     NGIDMDISGIPIPVCSCTGAPQQCYRWGSGGWQSACCTTGMSIYPLPMSTKRRGARIAGRKMSLGAFKKVLEKLAAEG-YNFSNPIDLRTHWAKHGTNKFVTIR
LjBBR/BPC1     NGIDMDISSIPIPVCSCTGAPQQCYRWGSGGWQSACCRTGMSMYPLPMSTKRRGARIAGRKMSIGAFKKVLEKLAAEG-YNFSNPIDLKTYWTKHGTNKFVTIR
```

Group IB

```
AtBPC2         NGVSMDISGLPVPICTCTGAPQQCYRWGCGGWQSACCTTNISMHPLPMSTKRRGARISGRKMSQGAFKKVLEKLASDG-FNFGNPIDLKSHWARHGTNKFVTIR
AtBPC3         NGVSMDIGGLPVPVCSCTGMPQQCYRWGCGGWQSACCTTNVSMYPLPVNTKRRGARIAGRKMSQGAFRKVLEKLSSDG-FDFSNPIDLKSHWARHGTNKFVTIR
AtBPC1         NGVSMDISGLPVPVCTCTGTPQQCYRWGCGGWQSACCTTNISVYPLPMSTKRRGARISGRKMSQGAFKKVLEKLSTEG-YSFGNAIDLKSHWARHGTNKFVTIR
LotuS_2        NGIDMDISGLPVPVCSCTGSPQQCYRWGCGGWQSACCTTNVSIYPLPMSIKRRGARIAGRKMSQGAFKKVLEKLAAEG-YNFANPIDLRTHWARHGTNKFVTIR
PtBPC1         NGIDMDISGIPIPTCSCTGTPQQCYRWGCGGWQSACCTTNVSVYPLPMSTKRRGARIAGRKMSQGAFKKVLEKLAAEG-YNFANPIDLRTHWARHGTNKFVTIR
PtBPC2         NGIDMDISGIPIPVCSCTGIPQQCYRWGCGGWQSACCTTNVSMYPLPMSTKRRGARIAGRKMSQGAFKKVLEKLAAEG-YNFANPIDLRTHWARHGTNKFVTIR
SlBBR/BPC5     NGINMDISVIPIPICSCTGAAQQCYRWGCGGWQSACCTTNLSSYPLPMNVKRRGSRIAGRKMSLGAFKKVLEKLASEG-YNFSNPIDLKPHWARHGTNKFVIIR
AaBBR/BPC1     NGIDMDISGIPIPVCSCTGSPQQCYRWGAGGWQSACCTTTISMYPLPMSTKRRGARIAGRKMSQGAFKKVLEKLASEG-YNFANAIDLRTYWAKHGTNKFVTIR
AaBBR/BPC2     NGIDMDISGIPIPVCSCTGVQQQCYRWGSGGWQSACCTTTISMYPLPMSTKRRGARIAGRKMSQGAFKKVLEKLASES-YNFANAIDLRLHWAKHGTNKFVTIR
VvBPC1-like    NGIDMDISGIPIPVCSCTGNPQQCYRWGCGGWQSACCTTSISMYPLPMSTKRRGARIAGRKMSQGAFKKVLEKLAAEG-YNFANPIDLRRHWAKHGTNKFVTIR
NtBBR/BPC1     NGIDMDISGIPIPVCSCTGAPQQCYRWGCGGWQSACCTTSISMYPLPMSTKRRGARIAGRKMSQGAFKKVLEKLAAEG-YSFANPIDLRTHWAKHGTNKFVTIR
StBBR/BPC1     NGIDMDISVIPIPVCSCTGSPQQCYRWGCGGWQSACCTTSISMYPLPMSTKRRGARIAGRKMSQGAFKKVLEKLAAEG-YNFANPIDLRTHWAKHGTNKFVTIR
SlBBR/BPC1     NGIDMDISVIPIPVCSCTGSPQQCYRWGCGGWQSACCTTSISMYPLPMSTKRRGARIAGRKMSQGAFKKVLEKLAAEG-YNFANPIDLRTHWAKHGTNKFVTIR
```

Group IC

```
OSBBR-likeB    NGIDLDLSRIPTPICSCTGAPQQCYRWGAGGWQSACCTTTISTYPLPMSTKRRGARIAGRKMSHGAFKKVLEKLAGEG-YNLNNPIDLKTFWAKHGTNKFVTIR
OSBBR-likeC    NGIDLDLSRIPTRICSCTGAPQQRYRWGAGGWQSACCTTTVSTYPLPMSMKPRGARIAGRKMSHGAFKKVLEKLASEG-YNLNNPIDLKTFWAKHGTNKFVTIR
HvBBR          NGIDFDISRIPTPVCSCTGAPQQCYRWGAGGWQSACCTTSISTYPLPMNTKRRGARIAGRKMSQGAFKKVLEKLAGEG-YNLNNPIDLKTFWAKHGTNKFVTIR
Elaeis_1       NGIDLDISGIPTPICSCTGTPQQCYRWGVGGWQSACCTTSISMYPLPMSTKRRGARIAGRKMSQGAFKKVLEKLAGEG-YNLSNPIDLKTYWAKHGTNKFVTIR
```

Group ID

```
PinTaeBPC1     NGIFLIFQLCLFLFALALEWLNNVTGGGNGGWQSACCTTSISMYPLPMNPNRRGARVAGRKMSGGAFRKLLERLGAEG-FNLNYPIDLKSYWAKHGTNKFVTIR
PinSitBPC1     NGIVFDISAMPIPVCTCTGVAQQCYRWGNGGWQSACCTTSISMYPLPMNPNRRGARVAGRKMSGGAFRKLLERLGAEG-YNLNYPIDLKSYWAKHGTNKFVTIR
Pin_CO362400   LMEFFDISTMPIPVCTCTGVAQQCYRWGNGGWQSACCTTSISMYPLPMNPNRRGARVAGRKMSGGAFRKLLERLGAEG-FNLNYPIDLKSYWAKHGTNKFVTIR
```

Group III

```
AtBPC7         DISSFDTSGVPPPVCSCTGVSRVCYKWGMGGWQSSCCTISISTYPLPMSTTRPGARLAGRKMSNGAYVKLLARLADEG-YDLSHPLDLKNHWARHGTNKFVTIK
PtBPC7         GKINFDPSGVPSPFCSCTGMPRVCYKWGAGGWQSSCCTDSISEHPLPMSSTRPGVRMAGRKMSNGAYVKLLLKLSAES-YNLSHPLDMKNHWARHGTNKFVTIK
SlBBR/BPC3     GRASADFSGLPQPFCSCTGVSRRCYK-CGGGWQSSCCTTSLSEYPLPFNPSKPGNRKAGRKMSNGAYNKLLCTLATEG-HDLSNPVDLKDHWAKHGSNKFITLK
```

Group II

Group IIA

```
AtBPC6         NQVVYDETTMPPPVCSCTGVLRQCYKWGNGGWQSSCCTTTLSMYPLPALPNKRHARVGGRKMSGSAFNKLLSRLAAEGHHDLSNPVDLKDHWAKHGTNRYITIK
PtBPC6         NQVAFDETTMPAPVCSCTGVFRQCYKWGNGGWQSSCCTTTLSMYPLPAVPNKRHARVGGRKMSGSAFSKLLSRLAAEG-QDLSNPVDLKDHWAKHGTNRYITIK
VvBPC6-like    NQVTFDESTMPAPVCSCTGVLRQCYKWGNGGWQSSCCTTTLSMYPLPAVPNKRHARVGGRKMSGSAFNKLLSRLAAEG-HDLSIPVDLKDHWAKHGTNRYITIK
NtBBR/BPC3     NQVSFDESTMPVPVCSCTGDLRPSYKWGNGGWQSSCCTNNLSMYPLPMLPNKRHARIGGRKMSGSAFTKLLSRLAAEG-HDLSNPVDLKDNWAKHGTNRYITIK
StBBR/BPC3     NQVAFDETTMPVPVCSCTGVLRPCYKWGNGGWQSSCCTTNLSMYPLPAVPNKRHARIGGRKMSGSAFTKLLSRLAAEG-HDLSNPVDLKNNWAKHGTNRYITIK
SlBBR/BPC4     NQVAFDETTMPVPVCSCTGVLRPCYKWGNGGWQSSCCTTNLSMYPLPAVPNKRHARIGGRKMSGSAFTKLLSRLAAEG-HDLSNPVDLKNNWAKHGTNRYITIK
```

Group IIB

```
AtBPC4         NLVTFDETTMPVPMCSCTGSTRQCYKWGNGGWQSSCCTTTLSQYPLPQMPNKRHSRMGGRKMSGNVFSRLLSRLSAEG-YDLSCPVDLKDYWARHGTNRYITIK
AtBPC5         VLVTFDEMTMPVPMCTCTGTARQCYKWGNGGWQSSCCTTTLSEYPLPQMPNKRHSRVGGRKMSGSVFSRLLSRLAGEG-HELSSPVDLKNYWARHGTNRYITIK
PtBPC3         NLINFDETTMTAPVCSCTGVPRQCYKWGSGGWQSACCTTTMSSYPLPQLPNKRRARVGGRKMSGNVFTRLLSRLAAEG-HDLSIPLDLKDYWARHGTNRYITIK
PtBPC4         NLINFDETTMPAPVCSCTGVPRQCYKWGSGGWQSACCTTTMSSYPLPQMPNKRHARIGGRKMSGNVFTRLLSRLAAEG-HDLAIPLDLKDYWARHGTNRYITIK
VvBPC4-like    NLVTFDESTMPVPVCSCTGVPRQCYKWGNGGWQSSCCTTTLSSYPLPQMPNKRHARMGGRKMSGSVFTRLLSRLAAEG-HDLSMPLDLKDYWAKHGTNRYIIIK
StBBR/BPC4     DQIQFDESTMPIPVCTCTGIPRQCYKWGSGGWQSSCCTTYLSEYPLPQLPNKRHARLGGRKMSGSVFSRLLTRFAVAD-RDLSMPIDLKTYWAKHGTNRYITIK
HaBBR/BPC1     DQISFDESTMPIPICSCTGVARQCYKWGSGGWQSSCCTTTLSVYPLPQMPNKRHSRMGGRKMSGTVFTRLIGRLASQG-HDLSAPVDLKNYWANHGTNRYITIK
```

Group IIC

```
Elaeis_2       NQVTFDEATMPTPVCSCTGKYQQCYKWGNGGWQSACCTTTLSMYPLPVMPNKRHTRMGGRKMSGGAFRKLLSRLAAEG-HDLLQPVDLKDHWAKHGTNRYITIK
OSBBR-likeD    NQVAFDDSTMPAPACSCTGKLRQCYKWGNGGWQSSCCTMNISMYPLPVMPNKRHARMGGRKMSGGAFTKLLSRLAAEG-HDLSTPVDLKDHWAKHGTNRYITIR
HvBBR_2        NQVSFDESSMPAPACSCTGVLRQCYKWGNGGWQSSCCTMSMSLYPLPVMPNKRHARMGGRKMSGSAFTKLLSRLAAEG-HDLSASGDLKDHWAKHGTNRVITIP
```

Group IID

```
SlBBR/BPC2     LKINFDDSAMPVPVCSCTGTPQPCYKWGHGGWQSACCTTTISMYPLPQISNKRYSRVGGRKMSGGAFSKLLNRLAAQG-YDLSIPLDLKDHWAKHGTNRYSTLK
StBBR/BPC2     FQINFDESAMPVPVCSCTGTPQPCYKWGHGGWQSACCTTTISMYPLPQISNKRYSRVGGRKMSGGAFSKLLNRLAAQG-YDLSIPLDLKDHWAKHGTNRYSTLK
NtBBR/BPC2     NQINFDESAMPVPVCSCTGTPQPCYKWGHGGWQSACCTTTISMYPLPQISNKRYSRVGGRKMSGGAFSKLLNRLAGQG-YDLSVPLDLKDHWAKHGTNRYSTLK
HaBBR/BPC2     NQVNFDETAMPVPVCSCTGVPQPCYRWGSGGWQSACCTTTMSMYPLPLVTNKRYSRVGGRKMSGGAFTKLLTRLASEG-YDLSTPLDLKDHWAKHGTNRYSKLK
AaBBR/BPC3     NQVNFDESAMPVPVCSCTGAPQPCYRWGSGGWQSACCTTTMSMYPLPQLANKRYSRVGGRKMSGGAFTKLLTRLSSEG-YDLSSQLDLKDHWAKHGTNRYSTVK
```

Group IIE

```
PinTaeBPC2     LPITFDASALPIPVCSCTGVPQQCYRWGSGGWQSACCTTSISMYPLPMNPNKRGARLGGRKMSGGAFAKLLARLIADG-YSLEYPVDLKNHWAKHGTNRYIRIQ
PinSitBPC3     NKITFDTSALPIPVCSCTGVPQQCYRWGSGGWQSACCTTSISIYPLPLNPNKRGARLGGRKMSGGAFAKLLARLIADG-YSLEYPVDLKNHWAKHGTNRYIRIQ
```

Group IV

```
PinSitBPC2     KSCVVYNPSMPIPYCSCTGANQPCYRWGNGGWQSACCTTMMSMYPLPMNPNKKGYRFPGRKMSAGAFQKLLVRLSLQG-IDVTKPIDLKEHWAKHGTNRYVTIK
Ceratopteris   YVPPYDLASTPVPFCSCTGTKRQCYRWGSGGWQSSCCTTFLSAYPLPVNHARRNSRVAGRKMSGGAFKKLLDRLAGTG-VDVTQPIDLKNHWAKHGTNKYVTVR
Adiantum       QKDLNFTVTTPIPYCSCTGSSQPCYRWGNGGWQSACCTNTLSVHPLPMNPLKRGYRLPGRKMSAGAFQKLVKRLTQEG-VDLSQPIDLKNYWAKHGTNRYITIK
Selaginella    AESSTTRKYYPAPFCSCTGTNQQCYRWGNGGWQSACCTTKISMYPLPMNPKKKGSRVAGRKMSAGAFMKLLDRLTAEG-VDVNSSVDLRPHWAKHGTNRYVTIK
Physcomitrella RIFRSQTVPTPIPYCSCTGMNQQCYRWGNGGWQSACCTTLISMFPLPLNPKKRGSRLAGRKMSAGAFDKLLEKLVSEG-VNINLPVDLREHWAKHGTNRYVTLR
Marchantia     EEIYVENVRYPIPHCSCTGVKQQCYRWGNGGWQSSCCTTMISMYPLPMNPTKRGSRLAGRKMSAGAFEKLLEKLALEG-VNVNYPVDLKDHWAKHGTNRYVTLR
```
